# Triboelectric Nanogenerators Based on Composites of Zeolitic Imidazolate Frameworks Functionalized with Halogenated Ligands for Contact and Rotational Mechanical Energy Harvesting

**DOI:** 10.1021/acsanm.4c06732

**Published:** 2025-02-18

**Authors:** Jiahao Ye, Tianhuai Xu, Jin-Chong Tan

**Affiliations:** Multifunctional Materials & Composites (MMC) Laboratory, Department of Engineering Science, University of Oxford, Parks Road, Oxford OX1 3PJ, United Kingdom

**Keywords:** triboelectric nanogenerators, metal−organic frameworks, composite material, functionalization, electrospinning

## Abstract

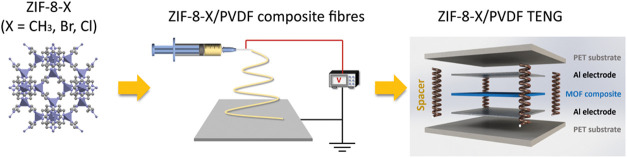

Triboelectric nanogenerator (TENG) based on the coupling effect of triboelectrification and electrostatic induction can convert mechanical motions into electric energy. Recent studies have found that metal–organic framework materials are promising triboelectric materials due to their large surface area and excellent tunability. In this study, we incorporated isostructural zeolitic imidazolate frameworks, ZIF-8-X (X = CH_3_, Br, Cl), into poly(vinylidene fluoride) (PVDF) electrospun fibers and assembled them in TENG devices to investigate the underlying relationship between functional group electronegativity (via varied imidazolate linkers) and triboelectric output performance. Results show that ZIF-8-Cl/PVDF composite fiber demonstrated the highest average voltage and current output of 312.4 ± 2.0 V and 4.90 ± 0.07 μA, respectively, which are 3.8 and 5.5 times higher than that of the pristine PVDF. The practicality of ZIF-8-X-based TENG was tested for harvesting energy from oscillatory motions to power up LEDs and capacitors. A freestanding mode TENG based on ZIF-8-Cl was also designed to harvest rotational energy without physical contact for wider applications. The working mechanism of ZIF-8-X-based TENG was also revealed through nanoscale-resolved chemical studies, providing valuable insights into the design of MOF materials for improved performance of TENGs.

## 1 Introduction

In the era of the Internet of Things (IoT), there has been an increasing demand for small-scale, mobile sensors. By 2030, more than 100 billion sensors are projected to be connected to the IoT with the electronics industry expected to grow into a USD 301 billion market in 2028.^1,2^ However, the energy supply for this enormous number of sensors could be a big challenge. Current technologies for energy transfer and storage will inevitably raise serious environmental concerns and maintenance difficulties.^3,4^ Therefore, triboelectric nanogenerator (TENG), an evolving energy harvesting technology, has drawn tremendous attention from researchers for its outstanding ability to convert mechanical motions into electrical energy under a variety of scenarios.^5−12^

Metal–organic framework (MOF), a class of porous materials that are fabricated by the coordination bond between organic ligands and inorganic metal ions, has diverse applications in gas absorption,^13^ drug delivery,^14^ catalysis,^15^ and sensing.^16^ Recently, various studies have explored the potential of using MOF for triboelectric nanogenerators.^17−23^ Attributed to their highly tunable structure and functional properties, MOFs have shown significantly improved TENG performance by enhancing charge generation capabilities and retaining device stability across diverse environmental conditions and applications.^24,25^ In 2019, Wen et al. introduced a copper-based MOF, HKUST-1, to improve the humidity resistance of the fabricated TENG devices due to the absorption of water molecules by HKUST-1 filler and subsequently higher dielectric constant.^26^ Following this, a fluorinated MOF was prepared by Guo et al. as a bifunctional filler to enhance the triboelectric performance of the device.^27^ Despite these promising advancements, a deeper understanding of the basic mechanisms and design principles of MOF-TENG systems is crucial for further development of this field.^28^

Recent studies have explored several strategies to enhance the triboelectric output of MOF-based TENGs, with three primary approaches being investigated: by modifying the framework topology,^29^ exchanging the metal ion center,^30^ and functionalizing the organic ligand.^31−33^ Among these strategies, ligand functionalization has emerged as a relatively simple and highly effective method. In 2022, Wen et al. modulated UiO-66 by introducing various functional groups onto the ligands and deposited them onto tin oxide electrodes. The triboelectric output performance of the UiO-66-X (X = H, NH_2_, NO_2_, Br) films are ranked in the order of NO_2_ > Br > H > NH_2_, which correlates to the electron-withdrawing nature of the functional group.^32^ In addition, Wang et al. reported a high-performance TENG device based on UiO-66-4F, where the enhanced charge generation property was attributed to the strong electron-withdrawing effect of the fluorinated group.^31^ While several studies have been conducted on the ligand functionalization approach, most of them have primarily focused on the UiO-66 system, which means that the universal applicability of this approach across different MOF structures remains unexplored. Given the critical role of functional groups in contact electrification,^34^ it is essential to develop a clearer understanding of the relationship between the end-group electronegativity and triboelectric output. In this research, we henceforth investigated the broader applicability of the functionalization approach by focusing on a well-studied zeolitic imidazolate framework (ZIF), ZIF-8. Recently, ZIF-8 has been characterized as a tribo-positive material with promising applications in energy harvesting.^35−40^ For example, Khandelwal et al. developed the first ZIF-8-based TENG device in 2019, demonstrating a sustainable triboelectric output of 164 V and 7 μA when contacting against Kapton. Moreover, Ma et al. prepared a TENG based on ZIF-8@ZnO as a self-powered methanol sensor. When paired with a tribo-negative PVDF film, the prepared TENG yields a triboelectric output of 58 V and 10 μA, representing 2.4 and 3.3 times enhancement compared to the ZnO-based TENGs, respectively. Despite extensive research on ZIF-8-based TENGs, their triboelectric output remains relatively low compared to other MOF-based devices,^24^ and its potential of being a tribo-negative material has yet to be demonstrated.

In this study, we report a general strategy for tailoring and boosting the triboelectric effect of ZIF-8-based TENGs through the ligand functionalization approach. Conventional ZIF-8 (herein designated as ZIF-8-CH_3_) has been functionalized with various halogenated groups, including bromine and chlorine groups to yield the ZIF-8-Br and ZIF-8-Cl structures with similar morphology and crystallinity as tribo-negative materials. By incorporating these filler materials into PVDF electrospun fiber, we successfully prepared high-performance nanogenerators with the average electric output of 312.4 ± 2.0 V and 4.90 ± 0.07 μA over 100 cycles achieved by ZIF-8-Cl-based TENG due to the strong electron-withdrawing ability of the chlorinated group. The triboelectric performance of prepared devices shows a consistent trend with the electronegativity of the functional groups, supported by nanoscale-resolved Kelvin probe force microscopy (KPFM) and nanoscale Fourier transform infrared spectroscopy (nano-FTIR) studies. The ZIF-8-Cl-based TENG shows high durability over 40,000 cycles and demonstrates excellent practicability for real-world applications. The high-performance ZIF-8-Cl was further doped into PVDF membranes to build a freestanding mode TENG which further expands the applications of the MOF-based TENG, allowing energy harvesting of rotational energy without physical contact.

## 2 Methodology

### 2.1 Materials

All chemicals used in this study are commercially available. Zinc nitrate hexahydrate (Zn(NO_3_)_2_·6H_2_O), 2-methylimidazole (2-mIm), dimethylformamide (DMF), ethanol, and methanol were purchased from Sigma-Aldrich. 2-Bromo-1H-imidazole (2-Br-Im) and 2-chloro-1H-imidazole (2-Cl-Im) were purchased from Doug Discovery. HSV900 poly(vinylidene fluoride) (PVDF) was obtained from Arkema.

### 2.2 Synthesis of ZIF-8 and Its Halogenated Derivatives

The synthesis of the ZIF-8-X particles is adapted and modified based on previous reports.^41,42^

(1) For the synthesis of the ZIF-8, herein termed ZIF-8-CH_3_, 350 mg of Zn(NO_3_)_2_·6H_2_O (1.2 mmol) was first dissolved in 15 mL of DMF. After sonication for 5 min, 200 mg of 2-methylimidazole (2.5 mmol) was added to the solution and stirred for another 5 min for complete dissolution. Afterward, the solution was transferred to a 20 mL PTFE-lined stainless-steel autoclave and heated at 100 °C for 72 h. After cooling to room temperature, the resulting white suspension was centrifuged and washed three times with methanol to remove excessive linker and solvent. 70 mg of white ZIF-8-CH_3_ powder was harvested and activated at 70 °C.
(2) For the synthesis of ZIF-8-Br, 121 mg of Zn(NO_3_)_2_·6H_2_O (0.4 mmol) and 2-bromo-1H-imidazole (120 mg, 0.8 mmol) were dissolved in 4 mL of ethanol.^43^ Then, the solution was transferred to a 20 mL PTFE-lined stainless-steel autoclave and heated at 100 °C for 72 h. Then, the same reaction protocol and washing process were followed to yield 60 mg of a yellowish powder.
(3) A similar procedure was followed to prepare ZIF-8-Cl. 121 mg of Zn(NO_3_)_2_·6H_2_O (0.4 mmol) and 2-chloro-1H-imidazole (120 mg, 0.8 mmol) were dissolved in 4 mL ethanol by stirring for 10 min. The synthesis and washing procedures were the same as previously followed by ZIF-8-Br. 50 mg of yellowish powder was obtained after drying.

### 2.3 Fabrication of MOF/PVDF Composites

The MOF/PVDF composites were prepared by an electrospinning technique. The PVDF solution used for electrospinning was prepared by dissolving 13.7 wt % of HSV900 PVDF powder in DMF to form a polymer solution. The prepared ZIF-8-X (X = −CH_3_, −Cl, −Br) were then combined with the PVDF solution via mechanical mixing to yield a mass ratio of 1:19 between ZIF-8-X and HSV900 PVDF powder. The homogenized solutions were stored in a glass syringe and gradually released by a syringe pump at a rate of 0.15 mL/h through a nozzle (conductive blunt tip). During operation, the blunt tip was electrified at a voltage of 15 kV by a high-voltage generator, with an aluminum foil placed 16 cm underneath the nozzle, acting as the negative charge collector. These parameters were optimized based on our previous studies to achieve a stable Taylor cone at the electrospinning nozzle.^44,45^ After 1 h of electrospinning, the electrospun fibers forming a porous membrane were then peeled off and dried to obtain approximately a nominal thickness of 120 ± 8 μm. The nanofibers produced by the high voltage formed uniform composite fibers without obvious MOF aggregation. The fibers are subsequently cut into dimensions of 2 cm × 2 cm for the fabrication of TENG devices.

### 2.4 Testing of Contact-Separation Mode TENG

Aluminum foils of 2 cm × 2 cm in size were attached to the center of PET substrates with dimensions of 3 cm × 3 cm. Then, the electrospun membrane comprising MOF/PVDF composite fibers was sandwiched between a pair of Aluminum foils. Four TENG devices were prepared by PVDF, ZIF-8-CH_3_/PVDF, ZIF-8-Br/PVDF, and ZIF-8-Cl/PVDF. For a standard test, a prepared TENG device was vertically attached to the sample holder connected to a load cell (RS PRO). A permanent magnet shaker (Brüel & Kjær LDS V201) powered by a voltage-amplified arbitrary function generator (GW Instek AFG-2105) was operated on the other side of the TENG device to generate the contact-separation motion. To examine the triboelectric response of the prepared TENG under varying humidities, the setup was encapsulated in an enclosed glovebag. Humidified air was first introduced through a bubbler filled with water to increase the relative humidity (RH) up to 60%, ensuring no potential damage on the equipment. Subsequently, pure dry nitrogen gas was purged into the glovebag to gradually reduce the RH down to 10% while recording the triboelectric output of the TENG devices under contact-separation mode.

### 2.5 Testing of Freestanding Mode TENG

MOF/PVDF composites comprising ZIF-8-Cl for the freestanding mode TENG were prepared by the doctor blade coating method. The ZIF/polymer solution mixture was dripped onto a glass substrate, which had a sharp doctor blade at a fixed distance above the surface. The blade is then moved in line with the surface to obtain a film with a uniform thickness of 100 μm. The freestanding mode was designed to study the energy harvesting ability of TENG devices under rotational motions. The setup comprises a stator and a rotor. The rotor was a 3D-printed fan blade-shaped substrate coated with the ZIF-8-Cl/PVDF membrane. A motor was connected to the substrate to generate and control the rotational motion of the rotor. The stator was designed with noncontacting inner and outer electrodes which allows the periodic displacement of active material along the two electrodes while it rotates. The rotational speed of the sample was measured with a tachometer (RS AT-8).

### 2.6 Characterization

A field-emission scanning electron microscope (FESEM LYRA3 GM TESCAN) was used to examine the surface morphologies of the prepared MOF and the MOF/PVDF composite materials. Energy-dispersive X-ray spectroscopy (EDS) was conducted on a microscope to evaluate the element composition of prepared samples. X-ray diffraction (XRD) was performed on a Rigaku MiniFlex with a Cu Kα source (1.541 Å) to determine the crystallinity of the samples. Atomic force microscopy (AFM) height topography and nano-FTIR spectra of ZIF-8-X and composite fibers were characterized by a scattering-type scanning near-field optical microscope (Neaspec s-SNOM). Fourier transform infrared (FTIR) spectroscopy was performed with a Nicolet iS10 FTIR spectrometer equipped with an attenuated total reflectance (ATR) module. The far-IR spectrum was recorded at the multimode IR imaging and microspectroscopy (MIRIAM) Beamline B22 at the Diamond Light Source synchrotron. A Bruker Vertex 80v FTIR spectrometer equipped with an ATR accessory (Bruker Optics) was used to perform the measurement. The surface potential image was obtained in Kelvin probe force microscopy (KPFM) mode by using an Asylum Research Cypher AFM. The confocal Raman spectra were obtained by an Oxford Instruments WITec Raman microscope. The electrical outputs, including voltage and current, were measured by a digital oscilloscope (PicoScope 5444B) with a 100 MΩ high-voltage probe (Rigol RP1300H) and an electrometer (Keithley 6514).

## 3 Results and Discussion

**Figure 1:**
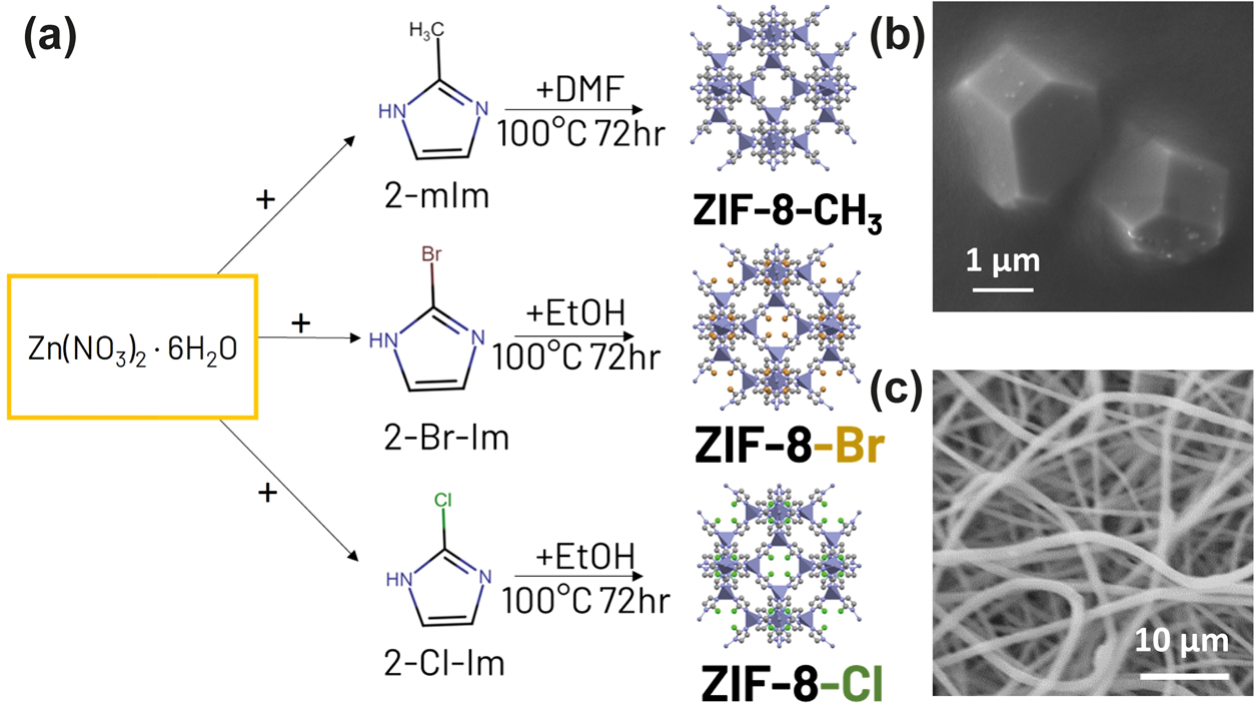
(a) Schematics illustrating the synthesis routes of ZIF-8-X and its halogenated derivatives, where X = −CH_3_, −Cl, −Br. (b) SEM micrograph of synthesized ZIF-8-Cl comprising micrometer-sized single crystals. (c) SEM micrograph of ZIF-8-Cl/PVDF fibers fabricated by electrospinning.

A group of functionalized ZIF-8-X materials was synthesized by a solvothermal method under the same reaction time and temperature but with different functional groups, namely, 2-mIm, 2-Br-Im, and 2-Cl-Im, as illustrated in Figure 1a. Figure 1b shows a representative SEM image of ZIF-8-Cl crystals, which displays an average particle size of ca. 2.1 ± 0.5 μm, with a similar morphology to those of other ZIF-8-X materials. The EDS elemental mapping analysis and additional SEM images for ZIF-8-CH_3_, ZIF-8-Br, and ZIF-8-Cl (Figures S1–S3, respectively) confirm the successful incorporation of halogenated groups. For TENG device fabrication and triboelectric performance testing, the prepared materials were then embedded into PVDF fiber by electrospinning, with the SEM image shown in Figure 1c. The photographic images of synthesized ZIF-8-X particles and the corresponding composites are shown in Figure S4.

### 3.1 Material Characterization of ZIF-8-X

**Figure 2:**
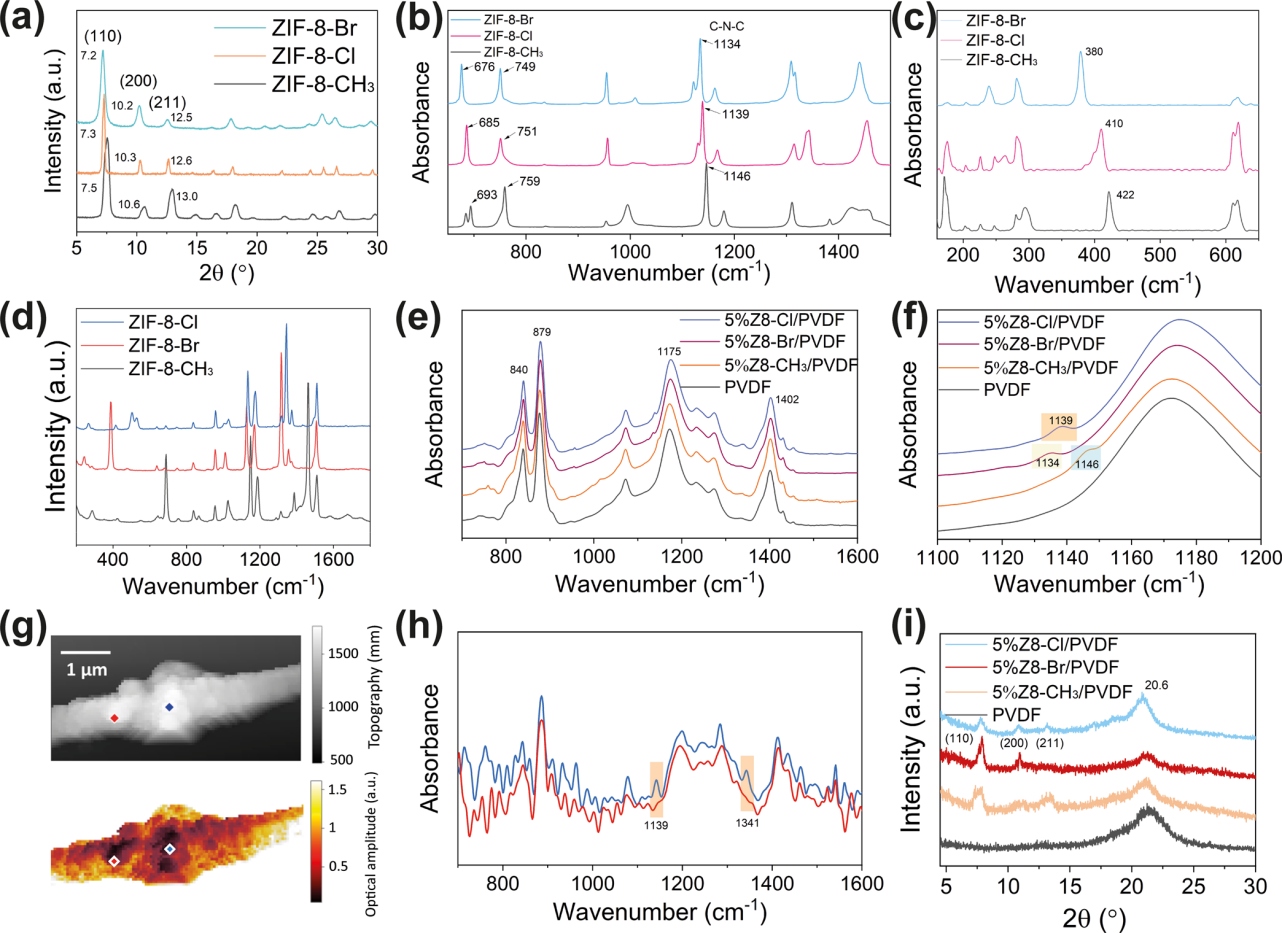
(a) XRD patterns of ZIF-8-X, where X = −CH_3_, −Cl, and −Br. (b) ATR-FTIR and (c) synchrotron far-IR spectra of ZIF-8-X nanoparticles. (d) Raman spectra of as-synthesized ZIF-8-X. (e) FTIR spectra of ZIF-8-X/PVDF composites, simplified as Z8-X/PVDF for all figures thereafter. (f) Superimposed FTIR spectra of ZIF-8-X/PVDF composites between 1100 and 1200 cm^–1^. (g) AFM topography of ZIF-8-Cl composite fiber (top) and its corresponding near-field IR absorption image (bottom). (h) Nano-FTIR spectra correspond to the red and blue points marked on the AFM image. (i) XRD patterns of the ZIF-8-X/PVDF composite fibers.

Figure 2a shows the XRD patterns of the synthesized ZIF-8-X samples, confirming their good crystallinity. All synthesized MOF crystals exhibit the (110), (200), and (211) facets, which indicate the formation of the sodalite (SOD) topology consistent with the simulated results, as shown in Figure S5. It was observed that the diffraction peaks of the (110) facet for ZIF-8-CH_3_, ZIF-8-Cl, and ZIF-8-Br are detected at 2θ of 7.54, 7.29, and 7.17°, respectively. This shift to the smaller diffraction angle means a larger porosity and lattice cell volume of the framework is formed due to the expansion of the unit cell structure by the bulkier end groups of the constituting ligand.^46,47^ Moreover, the XRD patterns of ZIF-8-X differ by the relative intensities at (110), (200), and (211) facets, as shown in Figure S5, which is due to the change in preferred orientation induced by the linker substitution.^48^ The synthesized ZIF-8-Xs also show good structural stability under ambient conditions and immersion in organic solvents, as illustrated in Figure S6.

In Figure 2b, the ATR-FTIR spectra of ZIF-8-X are similar despite some red shifts of vibrational modes as the linker gets bulkier and with weaker interactions.^49^ The shifts of peaks were observed at around 1140, 750, and 680 cm^–1^, where these IR bands are attributed to the imidazole ring vibrations due to different interactions between the end group and the imidazole ring. As the functional group of the framework gets heavier from CH_3_ to Cl and subsequently to Br, the vibrational frequency decreases and thus lowers the wavenumbers. The far-IR spectra shown in Figure 2c reveal the metal–ligand interactions in the terahertz region below ∼20 THz. Similar to ATR-FTIR, it can be seen that the collective mode attributed to the 12 THz peak (∼400 cm^–1^) systematically shifts toward a smaller wavenumber due to the larger framework. Figure 2d shows the Raman spectra of the ZIF-8-X particles. The spectrum for ZIF-8-CH_3_ matches well with previous reports,^50,51^ and the spectra for ZIF-8-X are similar in the range of 800–1800 cm^–1^ region, though a minor difference was detected due to the halogenation of the MOF. For ZIF-8-CH_3_, the characteristic peak at 686 and 1462 cm^–1^ was observed for C–H bond stretching and in-plane bending modes, respectively. For ZIF-8-Br, C–Br stretching occurs at 390 cm^–1^, and C–Cl stretching in ZIF-8-Cl takes place at 503 cm^–1^. The chemical bond vibrations of synthesized ZIF-8-X were also characterized by the near-field nano-FTIR technique, as shown in Figure S7. The height topography images of ZIF-8-X demonstrate the particle morphologies and nano-FTIR was taken on the MOF crystals with a 20 nm spatial resolution.^52^ The nano-FTIR spectra show broader peaks compared with ATR-FTIR due to the local measurements performed on isolated single-crystal MOFs, though the same redshift pattern is observed at ∼1150 cm^–1^ for the imidazolate ring stretching due to the bulkier functional group.

### 3.2 Material Characterization of ZIF-8-X/PVDF Composites

The synthesized ZIF-8-X are then incorporated into PVDF fiber by electrospinning technique as described in Section 2.3 with a schematic shown in Figure S8. The fiber form PVDF is preferred for the triboelectric nanogenerator application over the casted type PVDF due to a higher effective surface area between the fiber layers. Moreover, higher loading of MOF fillers can be embedded into the fiber without the formation of obvious aggregates due to the larger active surface area. The surface roughness of fiber also provides a higher contact area during the contact-separation process for triboelectric energy generation.^53^ The ATR-FTIR spectra of the prepared composite show the evolution of peaks from ZIF-8-X at ∼1140 cm^–1^, as displayed in Figure 2e,f, demonstrating the successful incorporation of ZIF crystals as a filler into the electrospun fiber matrix. The prepared composite fibers were further characterized by the nano-FTIR technique. The AFM topography of a ZIF-8-Cl embedded fiber in Figure 2g shows the successful incorporation of MOF. As denoted in Figure 2h, the nano-FTIR spectrum on the filler (blue) shows characteristic peaks at 1142 and 1342 cm^–1^ in addition to the neat PVDF matrix (red), which corresponds to the imidazolate ring stretching and aromatic C–N stretching in ZIF-8-Cl.^54^ The nano-FTIR characterizations for ZIF-8-CH_3_/PVDF and ZIF-8-Br/PVDF fibers are also shown and explained in Figures S9 and S10, respectively. The XRD pattern of the composite fiber shown in Figure 2i indicates the retained crystallinity of ZIF-8-X, showing minimal chemical interactions during the composite fabrication process. The dielectric properties of the prepared composite fibers were measured, and the results are shown in Figure S11. The dielectric constant of the fibers incorporating different MOF fillers exhibits only slight variations in the lower-frequency regime tested, which can be attributed to the low filler concentration and the high porosity inherent to the fibrous material. Although the dielectric constant is considered a crucial factor influencing triboelectric output, its impact has been controlled to isolate the effects of ligand modifications of MOF, ensuring the observed difference in triboelectric performance can be primarily attributed to the ligand modification.

### 3.3 Electrical Performance

**Figure 3:**
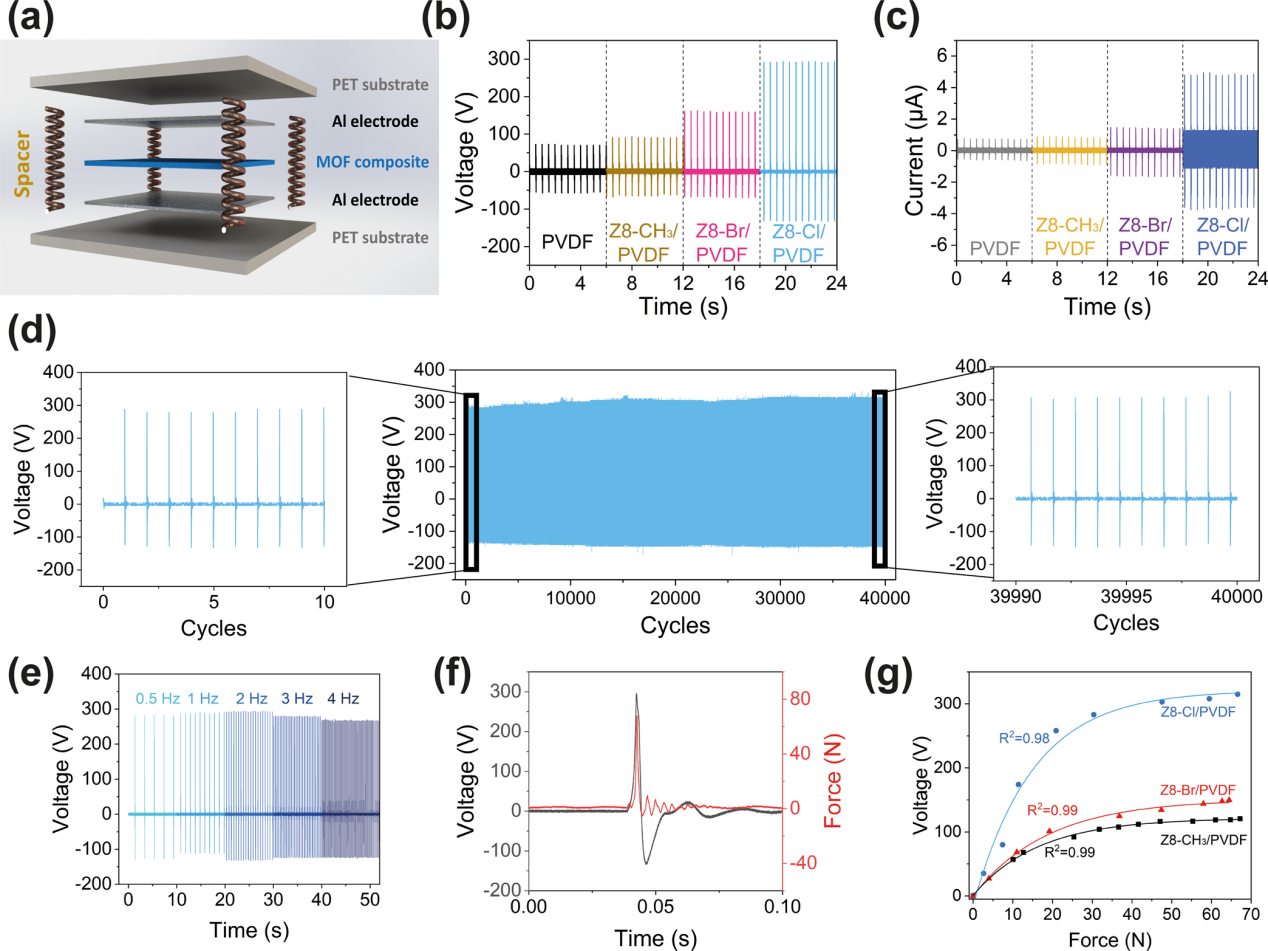
(a) Exploded view illustrating the stacked assembly of the prepared ZIF-8-X/PVDF TENG devices. (b) Open-circuit voltage output of the TENG devices. (c) Closed-circuit current output of the TENG devices. (d) Long-term durability of ZIF-8-Cl/PVDF-based TENG over a continuous running test of 40,000 contact-separation cycles. (e) Voltage output of ZIF-8-Cl/PVDF-based TENG under varying frequencies. (f) Relationship between the force experienced by the TENG device in a contact-separation cycle and the corresponding output current over the same time scale. (g) Voltage output of ZIF-8-X/PVDF-based TENGs under varying forces.

The prepared ZIF-8-X/PVDF composite fibers were cut and assembled into contact-separation type TENG devices for electrical performance testing. The fibers were sandwiched between aluminum electrodes and PET substrates, separated by sponge as spacer, as illustrated in Figure 3a. Photographs of the assembled device of the ZIF-8-X/PVDF-based TENG and the electrical performance testing rig are shown in Figure S12. The proposed working principle of the designed contact-separation type TENG for the ZIF-8-X-based composite fiber is summarized in Figure S13. In short, triboelectrification happens when ZIF-8-X/PVDF composite fiber contacts the Al electrode, causing electron transfer at the interface. During separation, electrostatic induction due to the potential difference drives electrons back to their original state. An AC output is generated through periodic contact and separation processes.

The electrical outputs of fabricated ZIF-8-X/PVDF fibers including open-circuit voltage and short-circuit current are measured and presented in Figure 3b,c, respectively. With the same MOF loading of 5 wt % into PVDF, ZIF-8-Cl/PVDF generates the highest average output of 312.4 ± 2.0 V and 4.90 ± 0.07 μA calculated from 100 cycles, which is 3.8 and 5.5 times higher than the neat PVDF fiber, followed by ZIF-8-Br and ZIF-8-CH_3_. For peak-to-peak voltage, each sample was tested for 100 cycles (as shown in Figure S14) and the average output and standard deviation were calculated and are displayed in Figure S15. The optimum loading of 5 wt % was selected based on the measured voltage output at various mass ratios of MOF fillers in the composites, as shown in Figure S16. This filler concentration was also supported by previous studies on PVDF-based composites,^55,56^ serving as a standard to evaluate the effect of ligand modification under consistent parameters. Based on the triboelectric output measurement of prepared ZIF-8-X/PVDF fibers, there is an obvious trend that their performance is markedly different according to the distinctive functional group of ZIF-8-X. To evaluate the effect of those halogen groups, the electrostatic potential maps of functionalized imidazole ligands are simulated using the density functional theory (DFT) by Gaussian 09W software,^57^ as displayed in Figure S17. Although the ligands will behave differently within a framework structure, as the nitrogen atoms are coordinated with the zinc ions, the simulation could provide insight into the electron-donating or -withdrawing effects of the functional groups. The methyl group of the 2-mIm linker demonstrates the only positive electrostatic potential on its surface, indicating its electron-donating nature, while 2-Br-Im and 2-Cl-Im exhibit strong electron-withdrawing effects due to the halogen substituents. As a result, the assembled conventional ZIF-8-CH_3_ with the methyl group linker has a partial positive charge with low electronegativity, making it less compatible with the high electron-withdrawing ability of PVDF by the presented fluorine groups. As a result, ZIF-8 is normally considered a tribo-positive material in recent studies.^39,40^ The minor improvement in the electrical output of ZIF-8-CH_3_/PVDF is attributed to the improved surface roughness through the introduction of MOF fillers. As the functional groups become more electronegative from −CH_3_ to –Br and to –Cl, both voltage and current output become higher due to the improved triboelectric charge generation property of the resultant material. For each TENG device, the collected voltage and current are relatively stable, with insignificant fluctuation of output. The triboelectric output of the prepared ZIF-8-Cl/PVDF fiber was evaluated against various materials, including copper, PDMS, Kapton, and poly(tetrafluoroethylene) (PTFE), with the corresponding voltage outputs presented in Figure S18. In general, the triboelectric output against aluminum and copper does not show significant differences due to the high electron-donating properties and high conductivity of metals. In contrast, as expected, the triboelectric output is lowered while pairing with other tribo-negative materials such as PDMS, Kapton, and PTFE. This trend aligns with the triboelectric series where materials with similar electron affinity exhibit lower charge generation when paired together. These findings further confirm the highly tribo-negative nature of the ZIF-8-Cl/PVDF composite.

To test the extended stability of the prepared samples, the same sample was tested under ambient temperature and humidity conditions, each with a 12-h interval. The result also shows good voltage output stability as shown in Figure S19. Moreover, the long-term durability of ZIF-8-Cl/PVDF-based TENG was also tested for over 40,000 cycles as demonstrated in Figure 3d. The prepared device maintained an excellent output voltage during long testing cycles, showing great potential for real-world applications. The SEM images of the fibers obtained before and after the durability test are demonstrated in Figure S20, showing minor changes in surface morphology. The sensitivity of prepared composites under various relative humidity (RH) conditions was also tested, as shown in Figure S21. The ZIF-8-Cl/PVDF composite fiber exhibited a relatively stable triboelectric output under low-humidity conditions, indicating its potential for applications in environments with controlled moisture. However, as the humidity increases, a 26% drop in the triboelectric output between 10% RH and 60% RH was observed. This reduction can be attributed to the increase in water adsorption onto the composite surface, which dissipates charge and reduces triboelectric charge generation. However, this 26% reduction of triboelectric output is not higher than that of the pristine PVDF (28%), suggesting that the incorporation of ZIF-8-Cl does not compromise the device performance under humid conditions.

The frequency dependency of the prepared TENG devices is also tested. From a testing frequency of 1–4 Hz, there is no significant change in the output voltage as the frequency varies for each type of TENG device, as shown in Figures 3e and S22. This is due to the same force applied to the material, and the same displacement has been achieved, such that each individual impact can be considered as discrete, independent of frequency. Small reductions of output voltage were observed at higher frequencies, which we attribute to the insufficient displacement during contact and separation. Previous research studied the relationship between the electrical output of TENG and stabilized force instead of the instantaneous peak force. In this case, we were able to measure the applied force and output voltage transiently and simultaneously using an integrated force sensor at one side of the TENG device during the contact-separation cycle. Figure 3f shows the corresponding relationship between current and force for the operation of a ZIF-8-Cl-based TENG recorded on the same time scale. It has been found that for all materials, each contact and separation process only happens within 0.05 s. The positive and negative currents from the current profile demonstrate the signal created during contact and separation, respectively. It is worth noticing that the appearance of current happens slightly before the force is applied, which is at the point when two materials are still approaching, and no physical contact has occurred yet. This finding supported the transfer of electrons as the mechanism during the contacting step. When the two triboelectric materials are approaching, the contact electrification process happens. However, the peak force and peak current occur at almost the same instance which implies that the current output has a strong correlation to the maximum force we applied. Once the applied force is retracted, the negative current shows due to electrostatic induction. The most negative current occurs slightly after the force is completely unloaded, as the triboelectric layers are recovered to their fully separated state. Similar results have been found in the other ZIF-8-X-based TENG devices that we studied, as shown in Figure S23. The relationship between the maximum instantaneous force and output voltage was then recorded by varying the voltage input to our electromagnetic shaker. The result reveals a nonlinear correlation between force and voltage, which can be characterized as an exponential relationship in that the sensitivity of measurement varies between the high- and low-pressure ranges. Figure 3g displays the voltage–force relationships for each of the TENG devices that we prepared under logarithmic fittings. The devices demonstrate a clear and predictable trend between output voltage and force, with *R*^2^ values of over 0.98, suggesting their potential use as highly sensitive pressure or force sensors. This nonlinear relationship originates from the presence of surface roughness on the surfaces of contacting materials.^58^ The real contact area between two materials will increase monotonically with the higher applied contact load due to more deformed material surface, until reaching a saturation at full contact based on Persson’s contact theory.^59^ As a result, the relationship between load and real contact area leads to the nonlinear, logarithmic load dependency observed in the triboelectric output.

### 3.4 Applications

**Figure 4:**
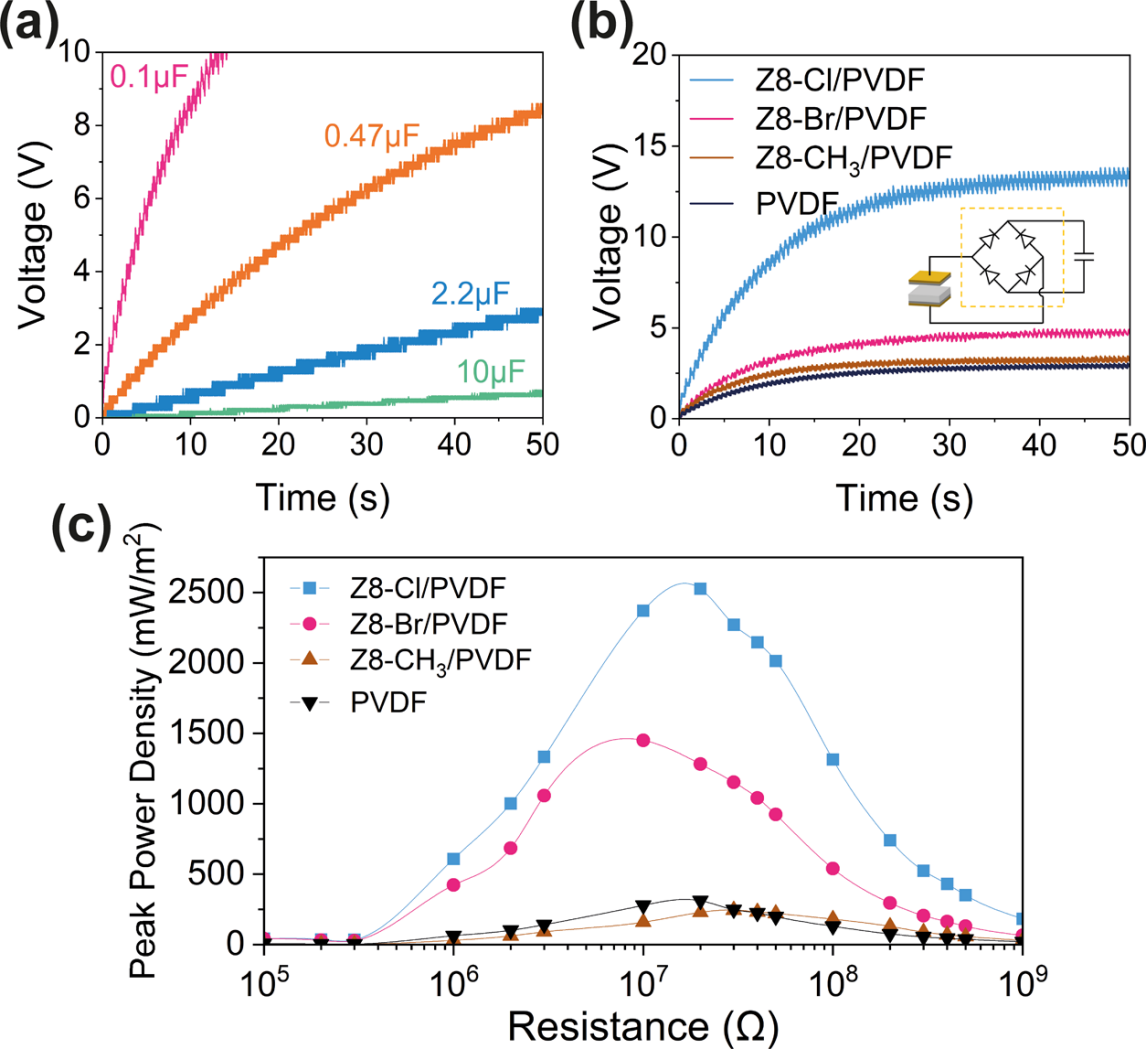
(a) Voltage profiles measured over different capacitors charged by the 2 Hz operation of the ZIF-8-Cl/PVDF-based TENG. (b) Comparison between charging speeds on a 0.1 μF capacitor by different ZIF-8-X-based TENGs. (c) Comparison between the peak power densities of prepared ZIF-8-X/PVDF TENG over a range of load resistances.

The alternating current produced by a TENG is harvested and stored in capacitors through a full rectifier circuit to convert it into direct current. Then, the rectified current can be used to charge small commercial electronics, such as calculators, LEDs, and capacitors. Here, several capacitors with capacitances ranging from 0.1 to 10 μF were charged by a steady 2 Hz oscillation motion on the ZIF-8-Cl/PVDF-based TENG, as shown in Figure 4a. The capacitors can be easily charged up to a reasonably high voltage for powering small electronics. The same experiment is done on other prepared TENG devices, and the results are shown in Figure S24. In addition, Figure 4b compares the capabilities of different TENG devices for charging a 0.1 μF capacitor. It is clear that the ZIF-8-Cl/PVDF-based TENG has exceptionally high charging ability compared with other materials, demonstrating excellent potential for energy harvesting. By calculation using the formula *E =* 1/2*CV*^2^, where *E* is the energy stored in a capacitor, *C* is the capacitance, and *V* is the voltage, we found that 22 times more energy is harvested in a 0.1 μF capacitor within 50 s compared with the pristine PVDF. The energy harvested through triboelectric displacement can be rectified to directly power LEDs.

The prepared TENG devices are then connected to an external resistor to form a closed circuit. Here, the closed-circuit voltage of each TENG is measured under different varying resistances from 100 kΩ to 2 GΩ. The power output *P* was calculated using the equation *P = V*^2^*/R*, where *V* is the closed-circuit voltage and *R* is the load resistance. Figure S25 summarizes the detailed correlation between the load resistance and power output for each of the prepared TENG devices. Similar to the capacitor charging correlation, the peak power of the ZIF-8-Cl/PVDF-based TENG is exterior to other devices, with an instantaneous power density of 2.54 W/m^2^, 8.2 times higher than PVDF, as shown in Figure 4c. The triboelectric output of the ZIF-8-Cl/PVDF-based TENG is on par with those of other PVDF-based TENG devices incorporating MOF or other filler materials under the contact-separation mode, as summarized in Table S1. Despite variations in the specific experimental configurations across different studies, the voltage, current, and power output of ZIF-8-Cl/PVDF-based TENG in this work demonstrate comparable performance to other high-performance TENG devices reported in the field.

### 3.5 Rotational Mechanical Energy Harvesting

**Figure 5:**
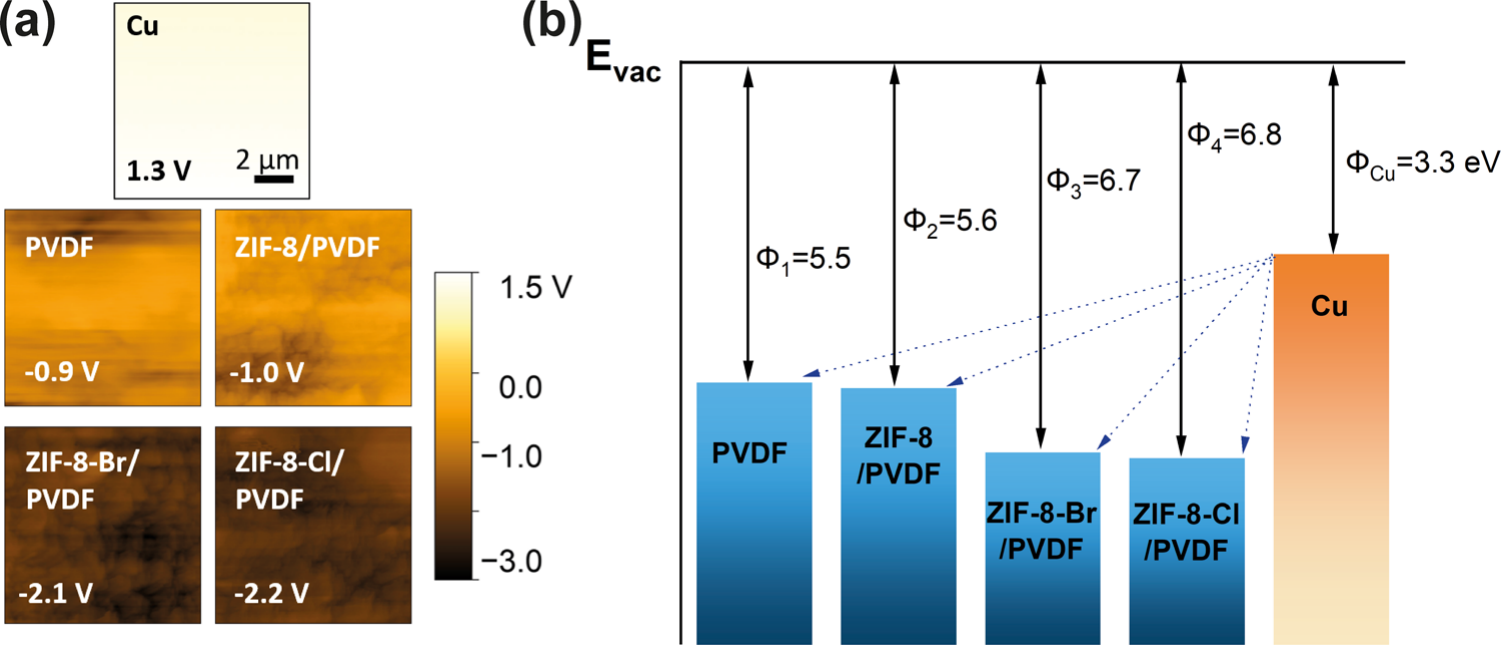
(a) Surface potentials of ZIF-8-X/PVDF composites and Cu electrode from KPFM measurements, presented under the same color scale. (b) Surface state model of ZIF-8-X/PVDF-based TENG. The work functions Φ were calculated based on the measured *V*_CPD_ value, using a Pt/Ir-coated AFM tip with a reported work function value of 4.6 eV.^63^

To demonstrate more practical applications of the prepared ZIF-8-Cl/PVDF composites, the membranes were also packed in a freestanding mode TENG, as described in Section 2.5, to examine the energy harvesting performance for rotational mechanical motions. Copper plates were used as electrodes in this configuration for their structural rigidity, processability, long-term stability under ambient conditions, and similar triboelectric output compared with aluminum electrodes. Under the designed freestanding mode, the rotor and stator operate without physical contact to minimize material wear. Therefore, the membrane needs to undergo prior triboelectrification by contacting a copper sheet before assembling into TENG, as the operation of the noncontact TENG relies on the residual charge on the material surface in the absence of contact triboelectrification. The KPFM results denoted an average surface potential of −2.2 V for ZIF-8-Cl-based PVDF film after prior contact, more negative than that of ZIF-8-CH_3_ and ZIF-8-Br, as presented in Figure 5a. The KPFM technique measures the contact potential difference (*V*_CPD_) of the tested materials, which provides insight into their relative work functions according to the following equation:
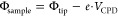
1where Φ_sample_ is the work function of the measured ZIF-8-X/PVDF composite, Φ_tip_ is the work function of the KPFM tip, and *e* is the elementary charge. The work function of a material represents the minimum energy required for the removal of an electron from a solid surface, which has been projected to play a critical role in the output performance of TENG devices.^60,61^ According to the electron transfer model, electrons transfer from a material with a lower work function to a higher one to maintain the Fermi level balance.^62^Figure 5b illustrates the surface state model according to the measured work functions. The energy levels of the functionalized composites are positioned according to the calculated work functions based on the measured *V*_CPD_ determined by KPFM. It is observed that as the functional group of ZIF-8-X changes to more electronegative halogens, the work function of the composites gets higher from 5.5 eV for the neat PVDF to 6.8 eV for the ZIF-8-Cl/PVDF. This increment enlarges the energy gap between the composite and copper. In order to balance the energy, the large energy gap facilitates the hopping of electrons upon contact, thereby generating more charge transfer during contact electrification.

The working principle of the noncontact TENG is illustrated and explained in Figure S26. For the freestanding mode TENG, two electrodes are positioned parallel to each other with an adjustable gap spacing between them and the freestanding dielectric layer (ZIF-8-Cl/PVDF composite membrane) is placed above the electrodes. Triboelectric charges will be induced on the surfaces of electrodes when the composite membrane is moving between the two electrodes, resulting in the flow of charges across the two electrodes through the external circuit.^64,65^ As a result, a freestanding mode TENG is a more flexible operating mode that does not require actual contact between the electrode and the dielectric material.

**Figure 6:**
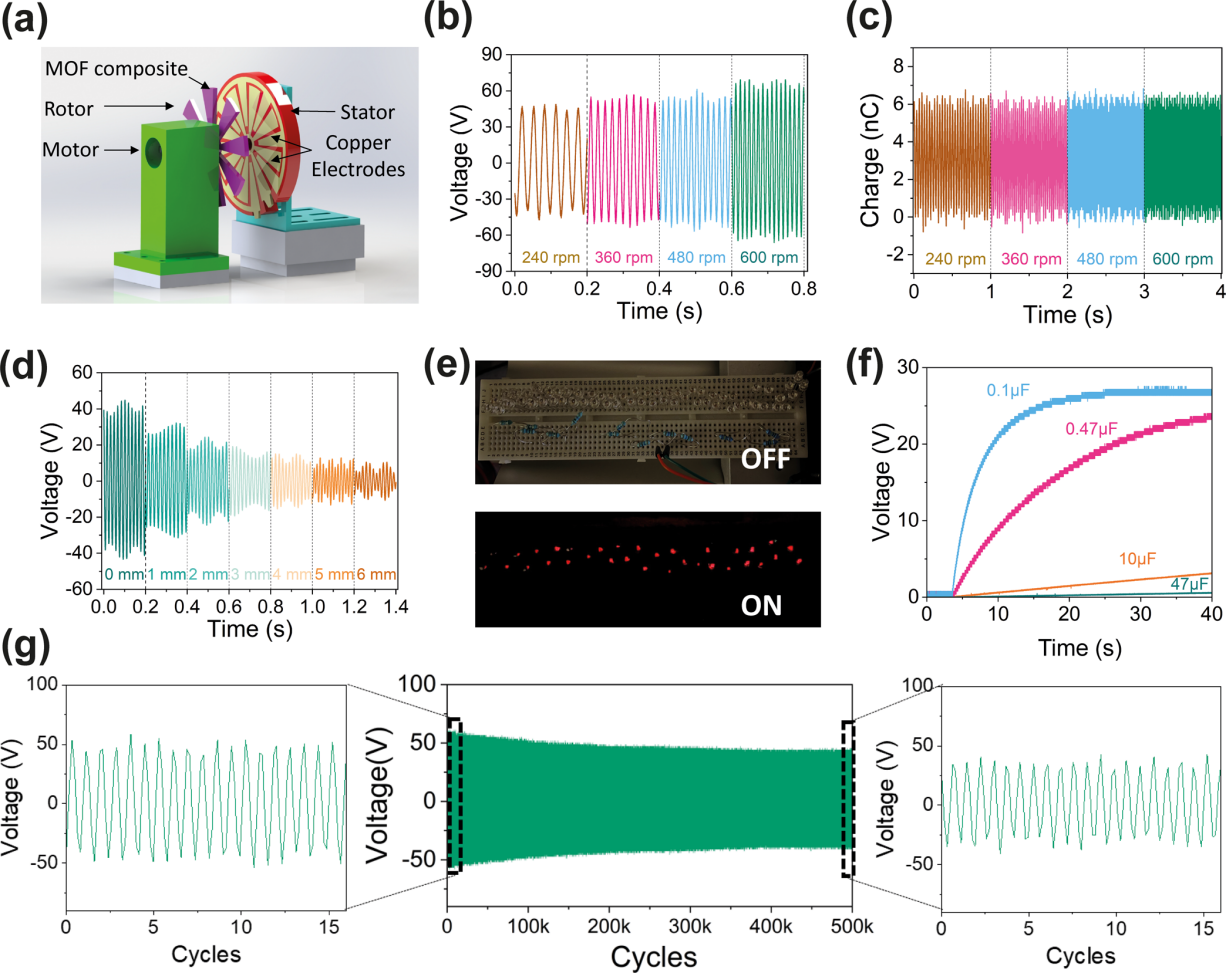
(a) Schematic diagram of noncontacting freestanding TENG setup. (b) Voltage output of ZIF-8-Cl/PVDF-based noncontacting TENG at varying rotational speeds. (c) Charge output of ZIF-8-Cl/PVDF-based noncontacting TENG at varying rotational speeds. (d) Voltage output of ZIF-8-Cl/PVDF-based noncontacting TENG with the same rotational speed but varying gap distance between the rotor and stator. (e) Continuous illumination of 40 LEDs by a ZIF-8-Cl/PVDF-based TENG. (f) Capacitor charging curves by operating ZIF-8-Cl/PVDF-based TENG at 600 rpm for charging the 0.1, 0.47, 10, and 47 μF capacitors. (g) Long-term durability of ZIF-8-Cl/PVDF-based noncontacting mode TENG measured over a continuous test comprising 50,000 cycles.

For the more efficient conversion of energy, we designed the inner and outer electrodes with an alternating pattern, as depicted in Figure 6a to enhance the electrical output. During operation, the rotation of active triboelectric material generates a consistent and sinusoidal voltage output, as shown in Figure S27. The design of 8 fan blades on the rotor and stator enables more frequent charge transfer, therefore increasing the AC output frequency. Figure 6b presents the open-circuit voltage of the noncontacting TENG at various rotational speeds, where the increase in rotational speed will improve the voltage output. The same trend is identified for the short-circuit current, as shown in Figure S28. However, it is worth noticing that the charge generated by the noncontacting TENG for each cycle remains constant regardless of the rotational speed as shown in Figure 6c. This is because no triboelectrification happens in noncontacting operational mode and a similar amount of charge is transferred at each cycle. The higher rotational speed increases only the frequency, not the quantity of charge transfer.

Furthermore, the current and voltage produced by a noncontact mode TENG can also be tuned by adjusting the distance between the MOF composite rotor and the electrode. In particular, the current and voltage reduce as the gap increases. By controlling the gap using the vernier stage, it has been observed that both voltage and current will experience exponential decay according to the distance between triboelectric layers, as shown in Figures 6d and S29. A similar degradation rate was found for the voltage and current after normalization (Figure S30). Since an AC output was generated by the rotational motion, harnessing this energy for practical applications like illuminating LED arrays or charging capacitors requires an additional step of rectification. This process was achieved through the circuit illustrated in Figure S31. Subsequently, the noncontacting TENG was employed to continuously illuminate LEDs due to its relatively high rotation speed (80 Hz), demonstrated in Figure 6e and Video S1, offering distinct advantages over contact-separation mode.^66^ In addition, the voltage across various capacitors when included in a circuit connected to the rotary device described is also measured in Figure 6f. The high operating frequency of the noncontacting rotary mode enables faster charging speed compared with the contact-separation mode, thereby offering great opportunities for efficient energy harvesting. The long-term output stability of the composite used in this mode was also tested, as depicted in Figure 6g, demonstrating a relatively high voltage production after 500,000 cycles. Although the absence of physical contact will lead to diminished surface charge over time and therefore a slight degradation of voltage output, the composite exhibits robust retention of triboelectric charges under ambient conditions.

## 4 Conclusions

In this work, we demonstrated an effective approach to enhance the triboelectric output of MOF-based materials by modifying their functional groups with higher electron-withdrawing capabilities. Through successful functionalization of ZIF-8 with halogenated groups to yield ZIF-8-Br and ZIF-8-Cl, we established a significant correlation between the electron-withdrawing ability of the functional group and the resulting output performance. Notably, our ZIF-8-Cl/PVDF composite fiber achieved remarkable voltage and current outputs of 312.4 ± 2.0 V and 4.90 ± 0.07 μA, respectively, which are 3.8 and 5.5 times higher than that of the pristine PVDF of the same nominal surface area. Moreover, the prepared ZIF-8-Cl-based device showed 8.2 times higher peak power density and demonstrated stability after 40,000 cycles. The origin of its high triboelectric performance has been revealed through molecular simulation and various nanoresolved characterization techniques including nano-FTIR and KPFM. In addition, practical applications of prepared TENG devices were tested by charging small electronics such as LEDs and capacitors. A rotational freestanding TENG device employing a ZIF-8-Cl/PVDF membrane further extends the practical use of the device, with promising applications in rotational energy harvesting. The proposed ligand halogenation approach, which introduces stronger electronegativity to the MOF, can significantly improve the charge-generating and trapping capability of the material and can be applicable to other tunable MOF structures. We believe this work not only provides valuable insights into the judicious design of MOF materials for improved performance of TENGs but also opens new possibilities for the application of MOFs in sustainable energy solutions.

## Data Availability

Data will be made available upon request.

## Supporting Information Available

X-ray diffraction, ATR-FTIR, nano-FTIR, AFM, and KPFM characterization data; voltage, current, and power output data; schematic diagrams of experimental setups; and device working mechanisms (PDF)
Demonstration of a noncontacting freestanding mode TENG based on ZIF-8-Cl/PVDF composite continuously powering an LED array (Video S1) (MP4)
