# Supplementary material for: Triboelectric Nanogenerators Based on Composites of Zeolitic Imidazolate Frameworks Functionalized with Halogenated Ligands for Contact and Rotational Mechanical Energy Harvesting
Source: ACS Appl Nano Mater. 2025 Feb 18;8(8):3942–53. doi: 10.1021/acsanm.4c06732 (PMC11877417; doi:10.1021/acsanm.4c06732)
Supplement: Supplementary file 1 — an4c06732_si_001.pdf [file an4c06732_si_001.pdf]

Supporting Information

for

Triboelectric Nanogenerators Based on Composites of Zeolitic Imidazolate Frameworks Functionalized with Halogenated Ligands for Contact and Rotational Mechanical Energy Harvesting

Jiahao Ye [a], *Tianhuai Xu* [a], and *Jin-Chong Tan* [a*]

[a] Multifunctional Materials & Composites (MMC) Laboratory, Department of Engineering Science, University of Oxford, Parks Road, Oxford OX1 3PJ, U.K.

* Corresponding Author and Lead Contact,

Email: jin-chong.tan@eng.ox.ac.uk

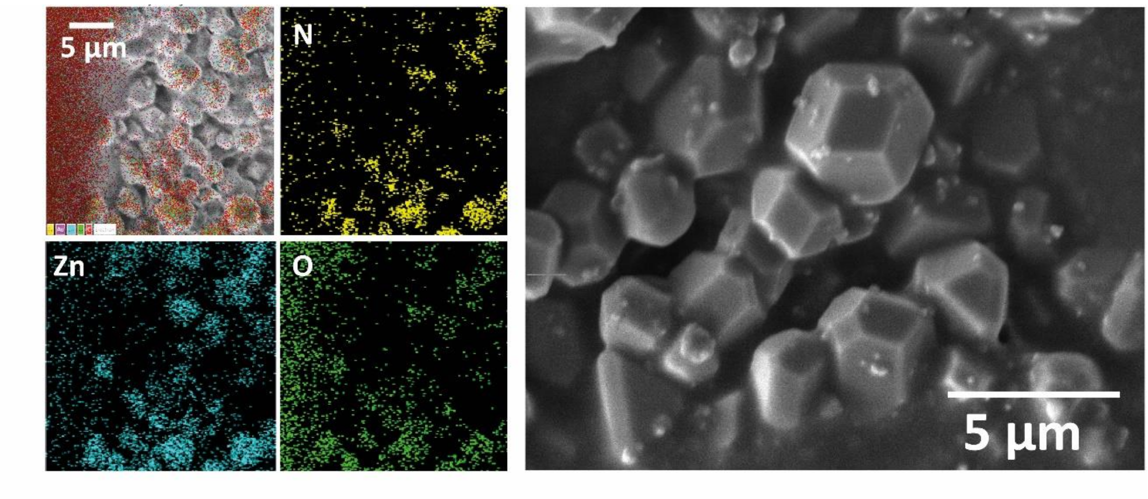

Figure S1. EDS elemental mapping analysis and FESEM image of ZIF-8-CH_3 crystals.

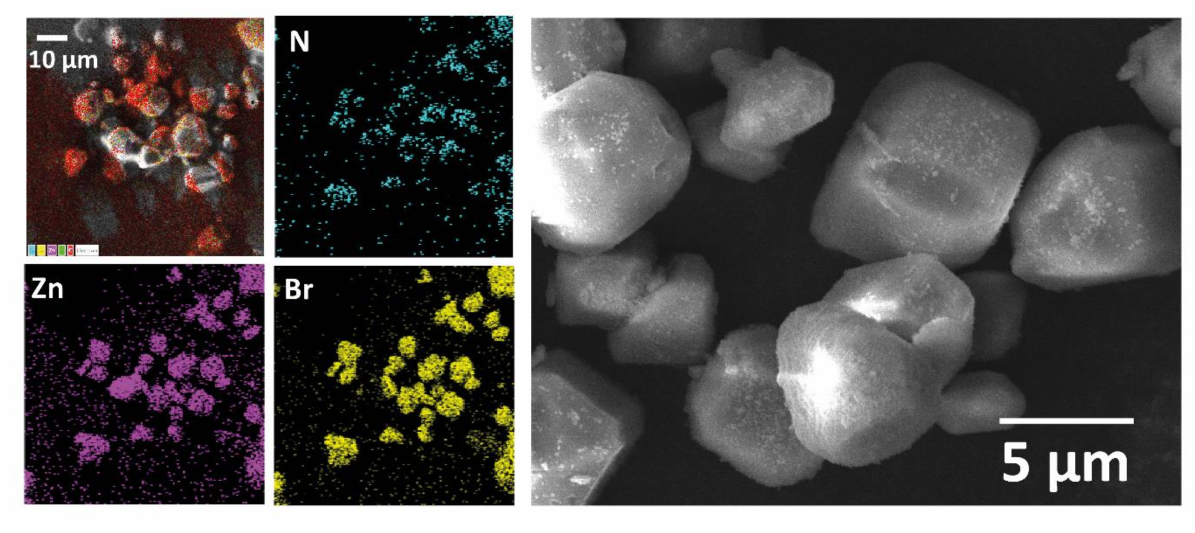

Figure S2. EDS elemental mapping analysis and FESEM image of ZIF-8-Br crystals.

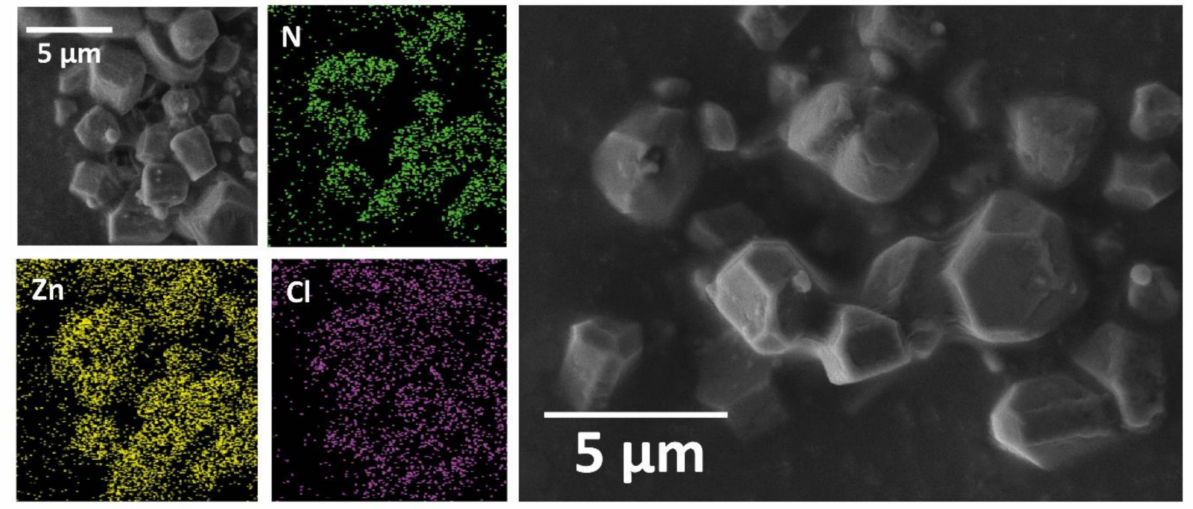

Figure S3. EDS elemental mapping analysis and FESEM image of ZIF-8-Cl crystals.

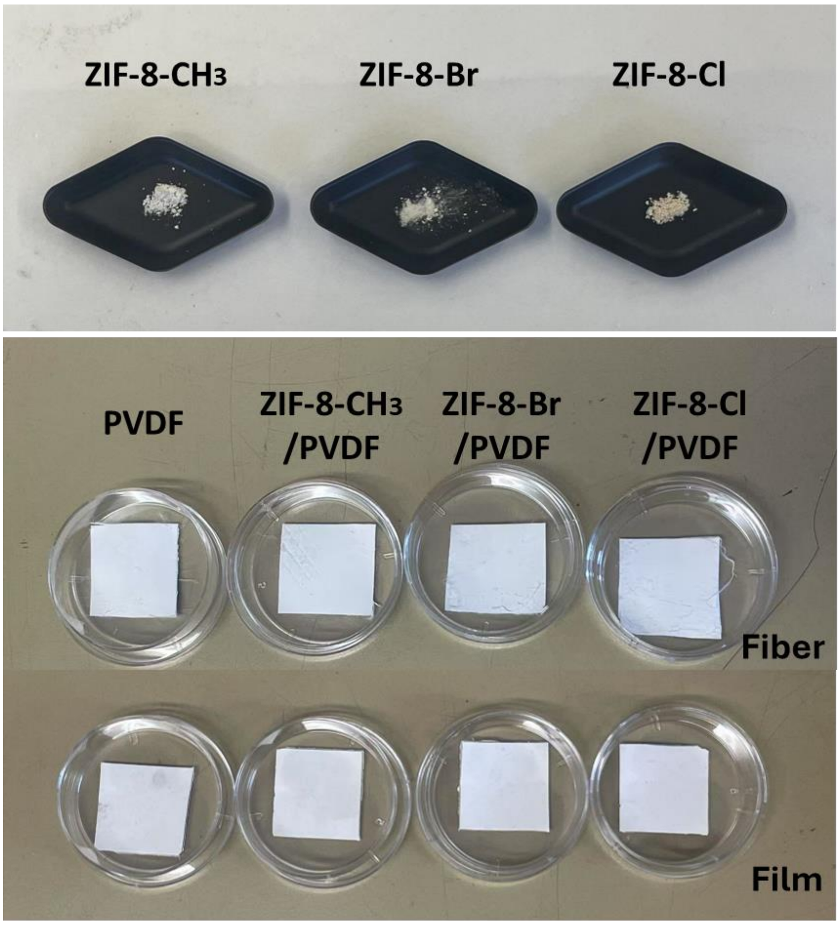

Figure S4. Image of synthesized ZIF-8-X crystals and fabricated composites.

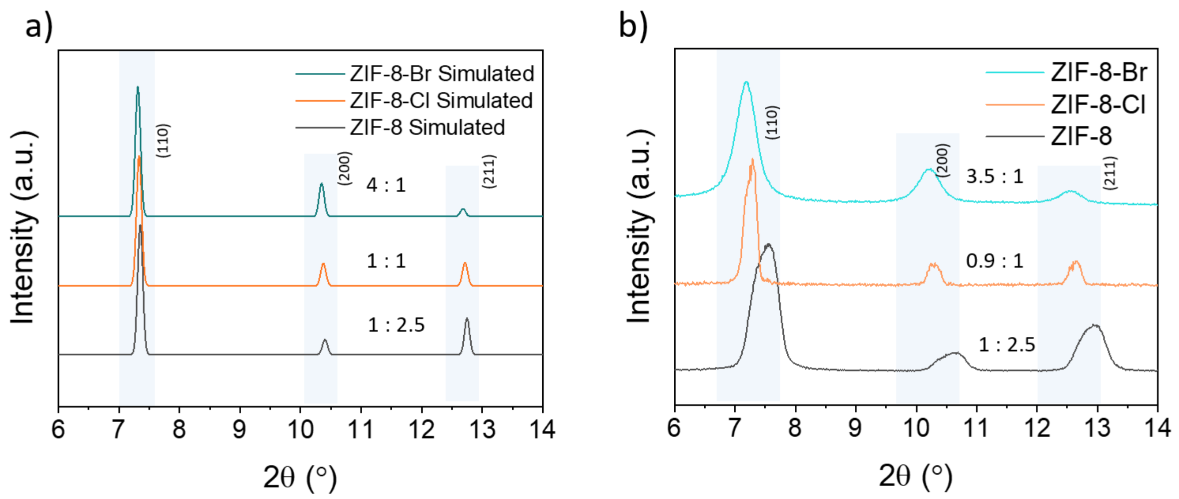

Figure S5. a) Simulated XRD patterns of ZIF-8-X where X = -CH_3, -Cl, -Br, and their relative peak intensities between (200) and (211) facets. b) XRD patterns of as-synthesized ZIF-8-X.

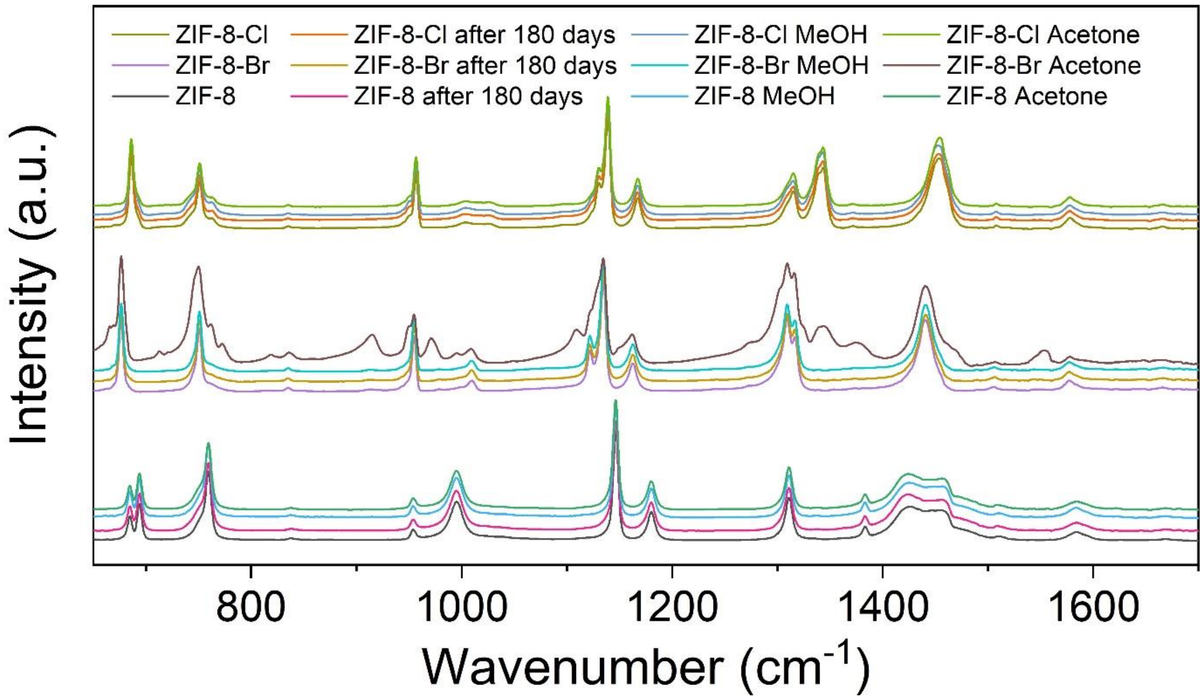

Figure S6. ATR-FTIR spectra of ZIF-8-X particles after exposure under ambient conditions for 180 days, and after being immersed in methanol or acetone solvents. All ZIF-8-X show good structural stability despite ZIF-8-Br showing a degradation in crystallinity after being immersed in acetone solution. Comparing with ZIF-8-Cl, ZIF-8-Br is less stable in polar aprotic solvent such as acetone due to the higher steric strain by larger size of Br atom and weaker electronegativity thus weaker bond with ZIF framework compare with Cl.

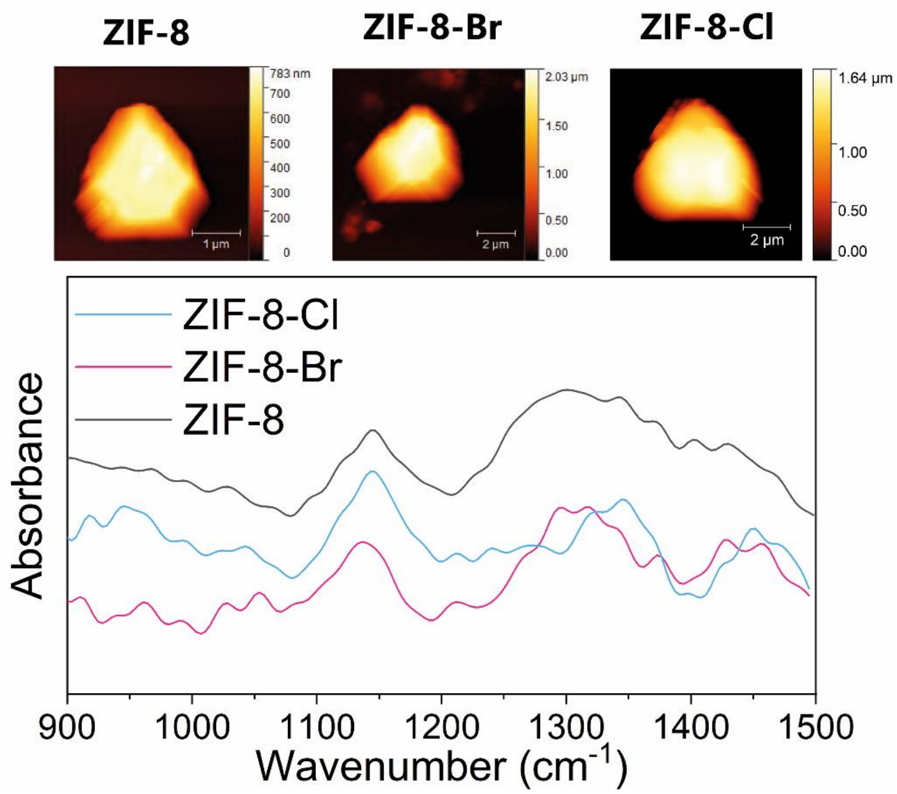

Figure S7. AFM topography and nano-FTIR absorption spectra of as-synthesized ZIF-8-CH_3, ZIF-8-Br, and ZIF-8-Cl.

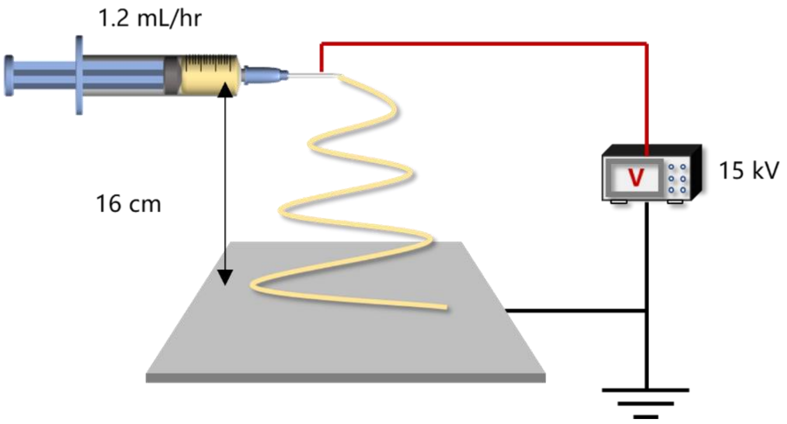

Figure S8. Schematic diagram of electrospinning process to produce ZIF-8-X/PVDF fibers. A 15 kV voltage is applied between the nozzle and the metal substrate with a height of 16 cm. The as-prepared ZIF-8-X/PVDF solutions were slowly dosed from the syringe at a rate of 1.2 mL/hr employing an automatic syringe pump.

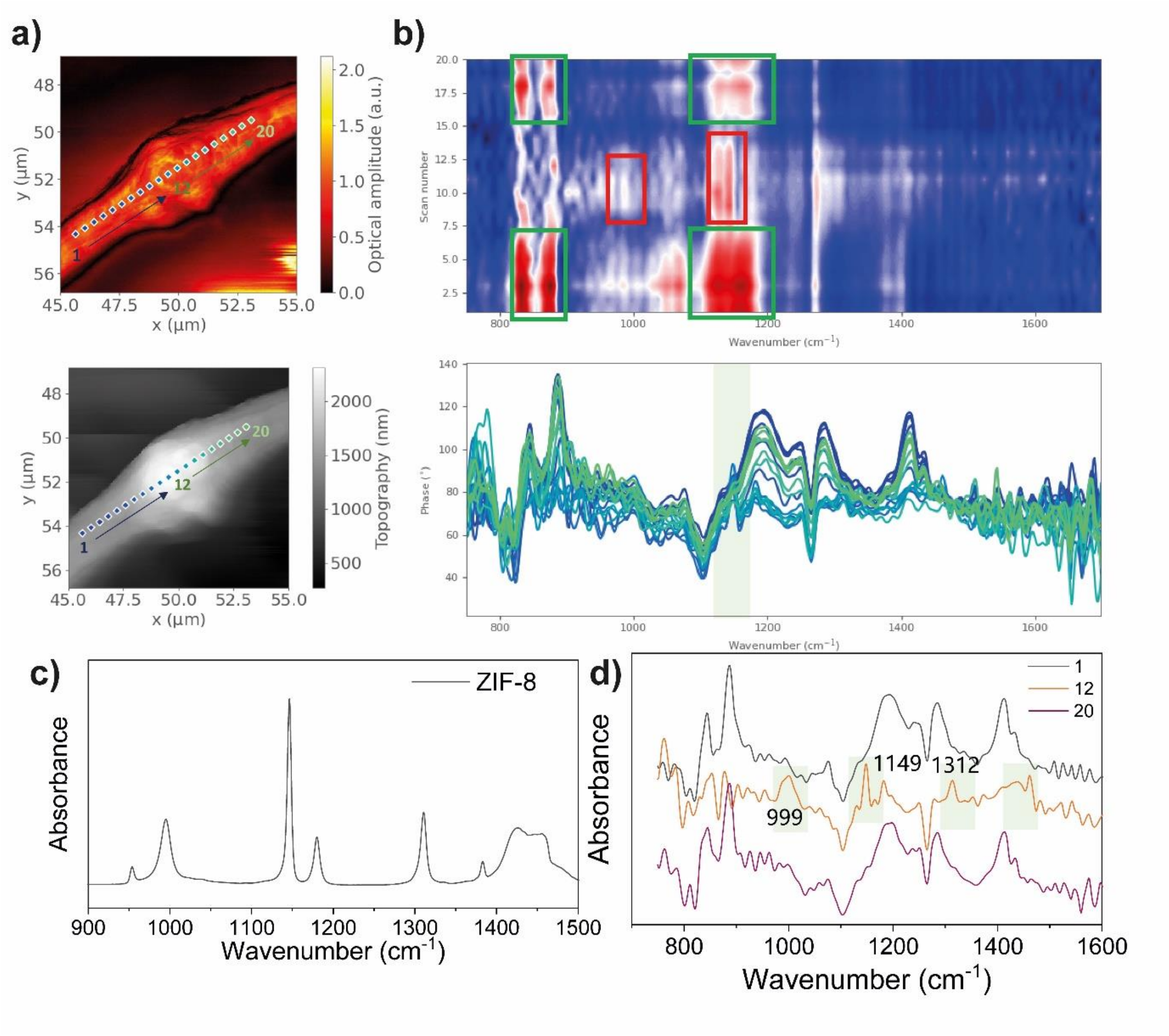

Figure S9. a) Nearfield infrared O2A signal and AFM height topography of the as-synthesized ZIF-8-CH_3/PVDF composite fiber. b) Contour plot and line-scan nano-FTIR spectra of ZIF-8-CH_3/PVDF composite along the fiber. The green box highlights the characteristic peaks of PVDF matrix, and the red box denotes the peaks for embedded ZIF-8-CH_3 particles. The FTIR peaks for the filler at 1000 cm^{-1} and 1150 cm^{-1} evolve as the scan progresses towards the center of the AFM scan. c) ATR-FTIR spectrum of ZIF-8-CH_3 particles. d) Nano-FTIR spectra of points 1, 12, and 20 along the line-scan. The peaks for ZIF-8-CH_3 at 999 cm^{-1}, 1149 cm^{-1} and 1312 cm^{-1} are distinctly observed at position 12 but not evident on the smoother part of the fiber.

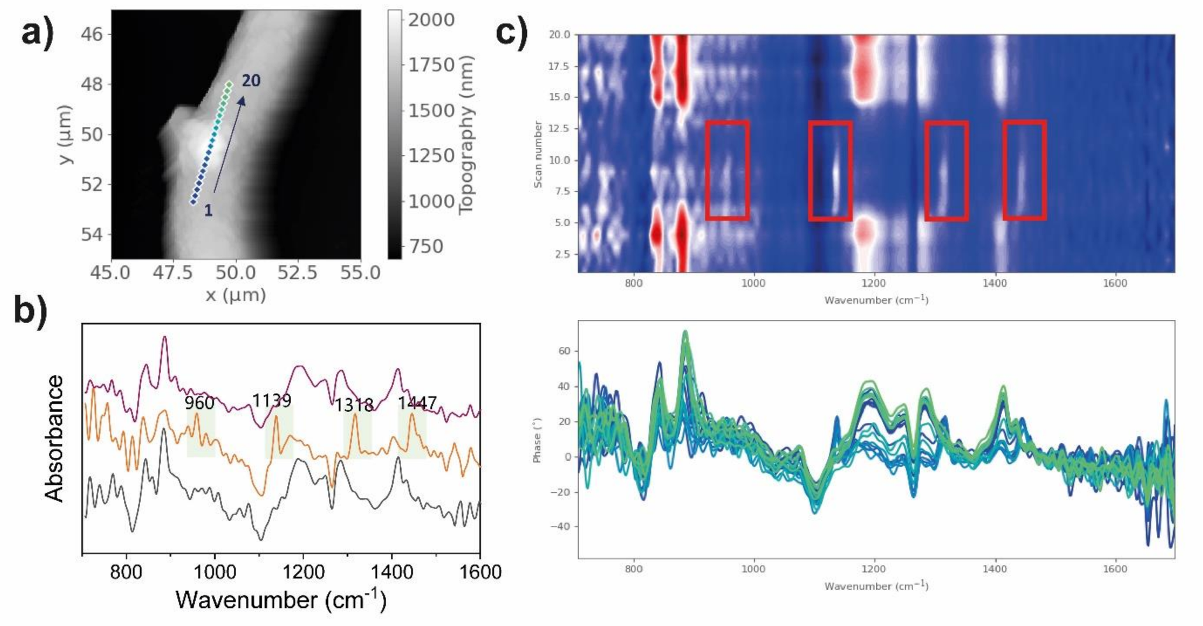

Figure S10. a) AFM topography of the as-synthesized ZIF-8-Br/PVDF composite fiber. b) Nano-FTIR spectra of points 1, 9, and 20 along the line-scan. The peaks for ZIF-8-Br at 960 cm^{-1}, 1139 cm^{-1}, 1318 cm^{-1}, and 1447 cm^{-1} are distinctly observable at point 9. c) Contour plot and line-scan nano-FTIR spectrum of ZIF-8-Br/PVDF composite along the fiber. The red box highlights the characteristic peaks of the embedded ZIF-8-Br particles as the scan progresses towards the center of the AFM scan.

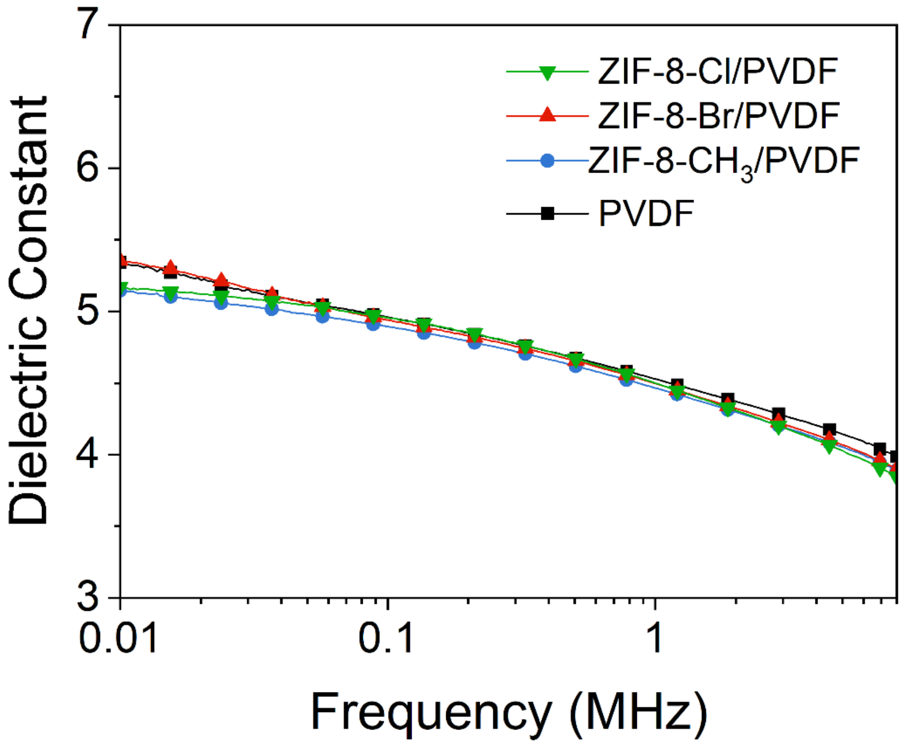

Figure S11. Dielectric constant of prepared PVDF-based composite fibers.

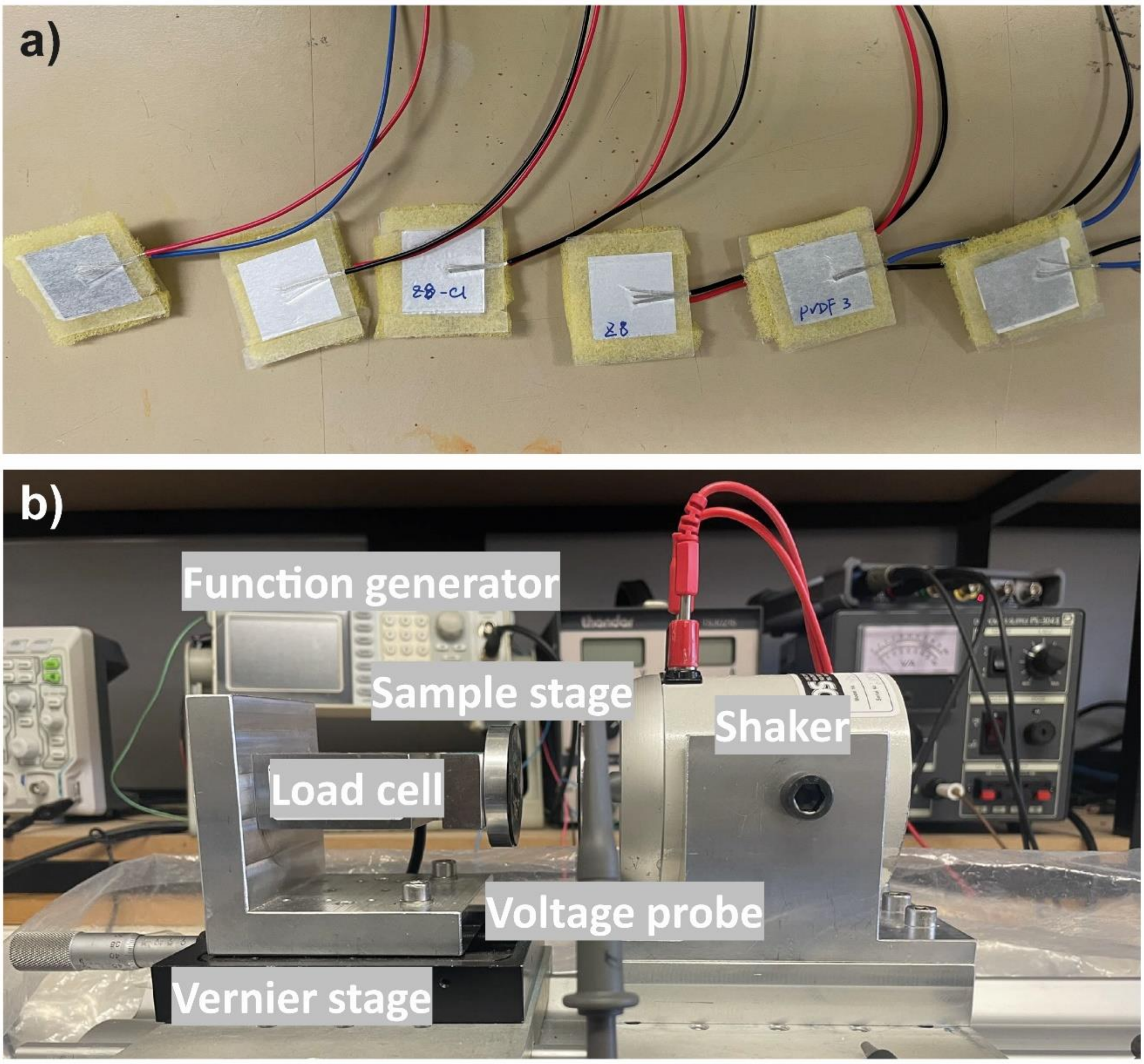

Figure S12. (a) Assembled ZIF-8-X/PVDF-based TENG devices. (b) Experimental setup for electrical performance measurement.

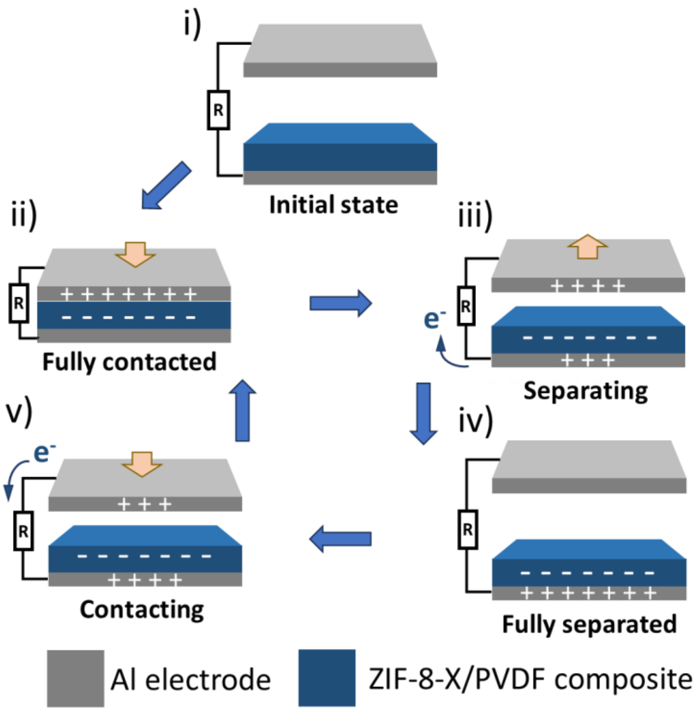

Figure S13. Schematic diagram of the proposed working mechanism (i-v) of a conductor-to-dielectric contact-separation mode TENG, based on the ZIF-8-X/PVDF nanocomposite.

i) Initially, there is no charge transfer between the Al electrodes as the top electrode is not in contact with the ZIF-8-X/PVDF nanocomposite film.

ii) Contact triboelectrification occurs when the top Al electrode is brought into contact with the ZIF-8-X/PVDF composite film. Equal and opposite charges are induced on the contacting interface. Tribo-negative ZIF-8-X/PVDF film carries negative charges.

iii) Upon release of the contacting force, a potential difference is induced between the top Al electrode and the composite film attached to the bottom Al electrode. Electrons are then driven to flow to the top electrode through the external circuit (R).

iv) When the contact force is completely released and a fully separated state is reached, the electron flow ceases, and an equilibrium is attained.

v) As the top electrode approaches the highly electronegative nanocomposite layer, this causes the electrons to flow in the reverse direction. The flow of electrons stops when a fully contacted state is reached in (ii), thereby completing the cycle.

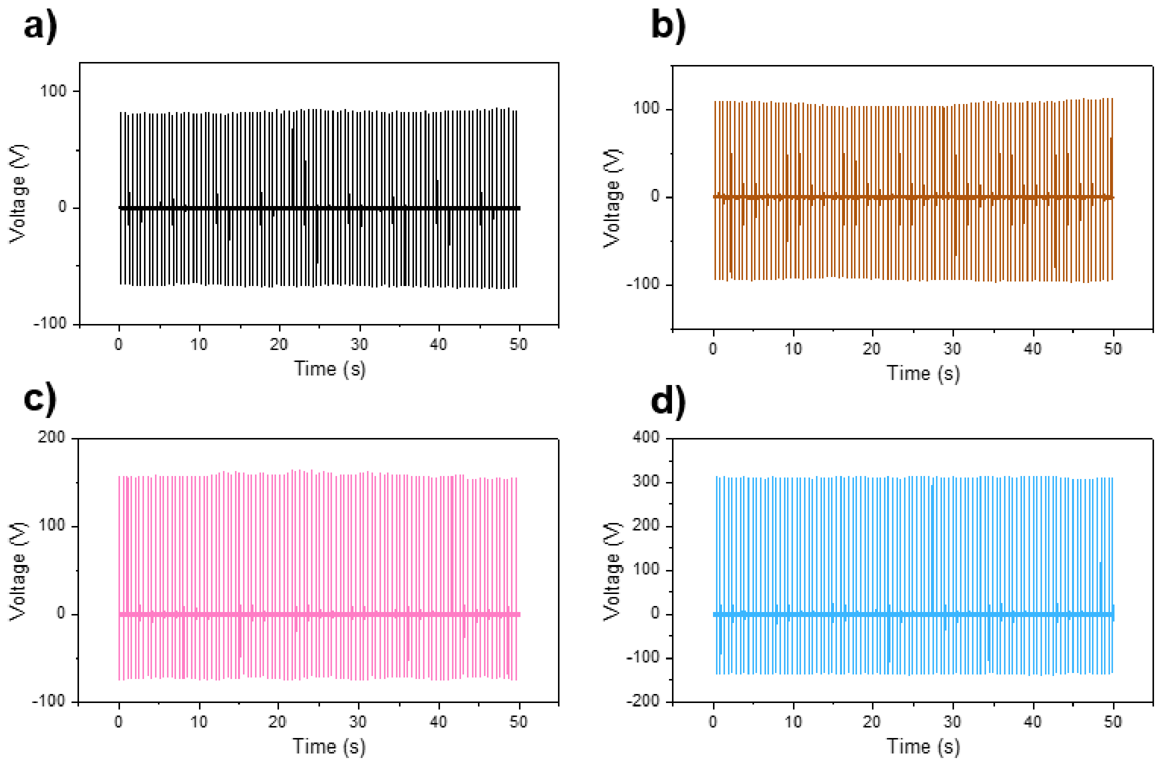

Figure S14. The voltage output over 100 contact-separation cycles generated by a) PVDF-based TENG, b) 5 wt% ZIF-8-CH_3/PVDF-based TENG, c) 5 wt% ZIF-8-Br/PVDF-based TENG, and d) ZIF-8-Cl/PVDF-based TENG.

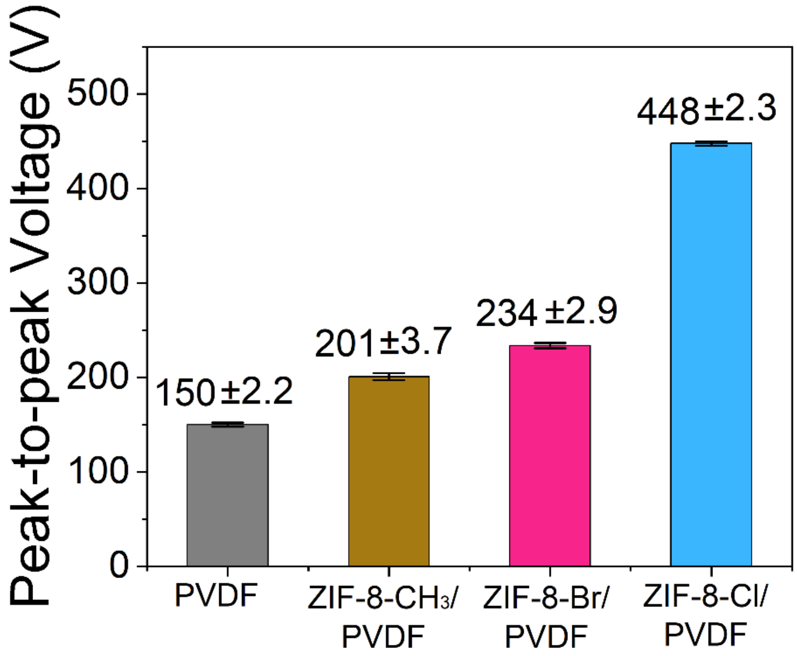

Figure S15. The average peak-to-peak voltage output generated by the 5 wt% ZIF-8-X/PVDF-based TENG. The mean and standard deviation values were determined from a total of 100 contact-separation cycles.

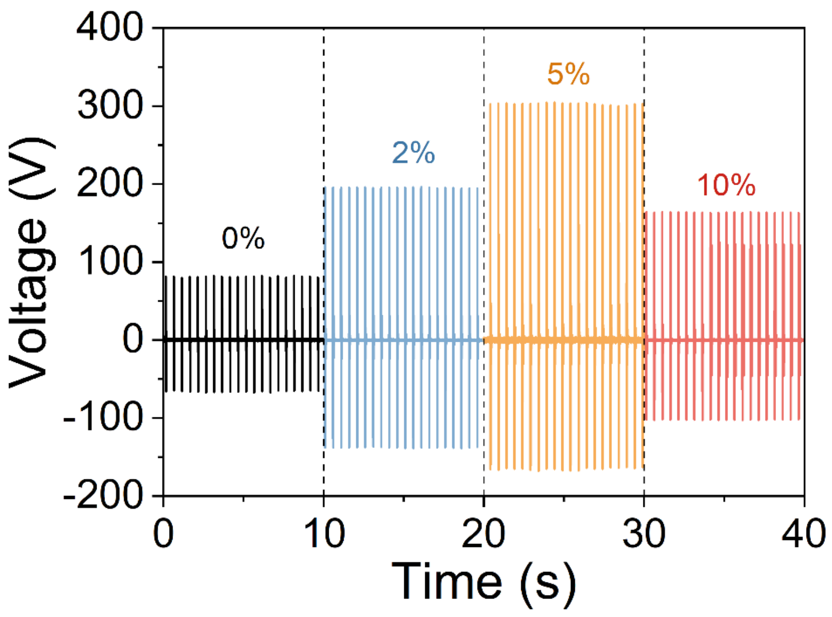

Figure S16. Open-circuit voltage of ZIF-8-Cl/PVDF based TENG at different mass loadings (wt %) under an oscillatory motion of 2 Hz.

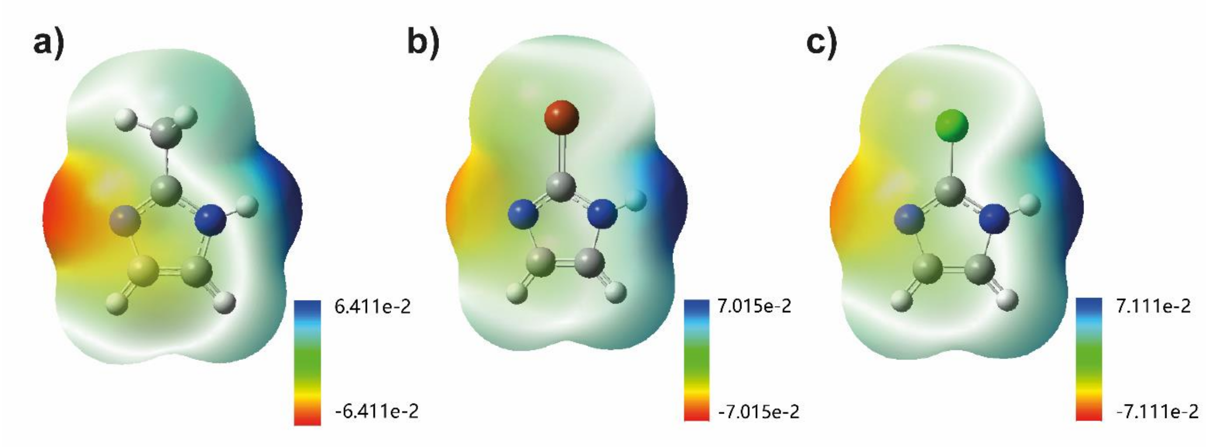

Figure S17. Electrostatic potential maps of (a) 2-mIm, (b) 2-Br-Im, and (c) 2-Cl-Im simulated by Gaussian 09W (unit=kcal/mol).

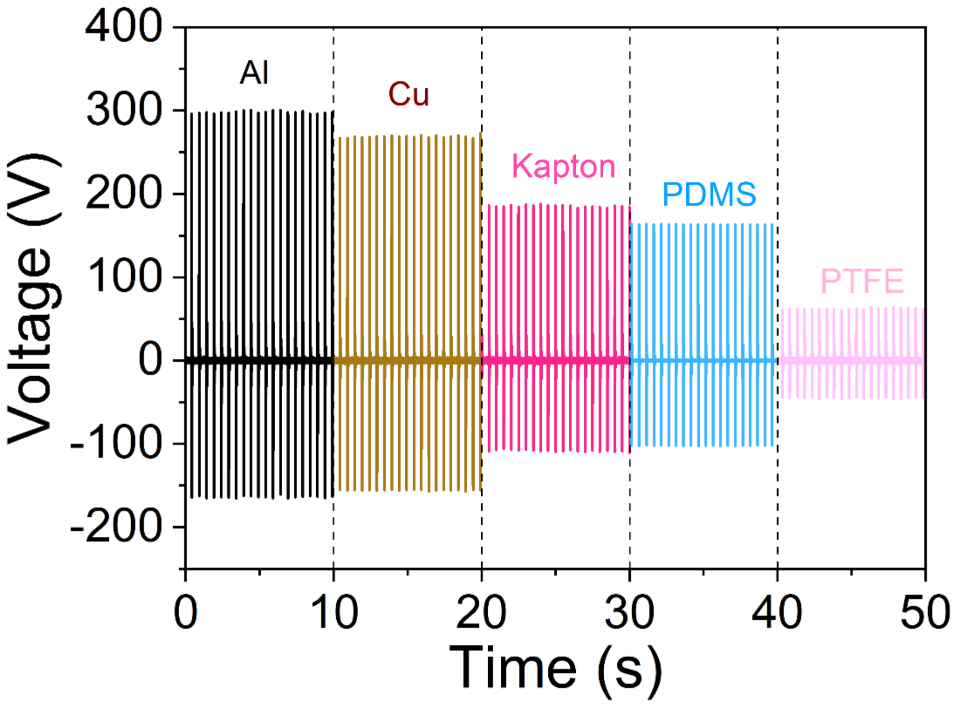

Figure S18. The voltage output of 5 wt% ZIF-8-Cl/PVDF fiber while pairing against different triboelectric materials.

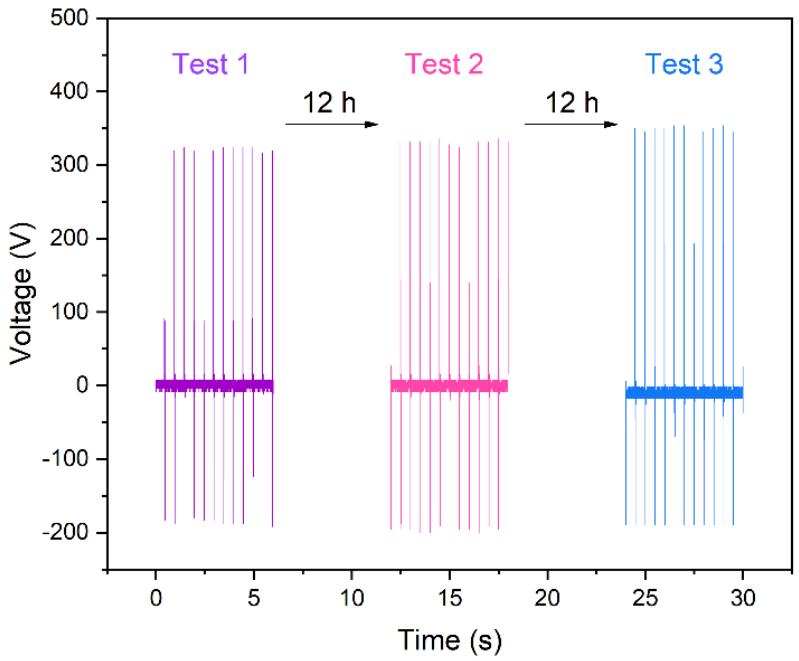

Figure S19. The voltage output generated by the 5 wt% ZIF-8-Cl/PVDF-based TENG under ambient conditions with a 12-hour interval between each set of tests.

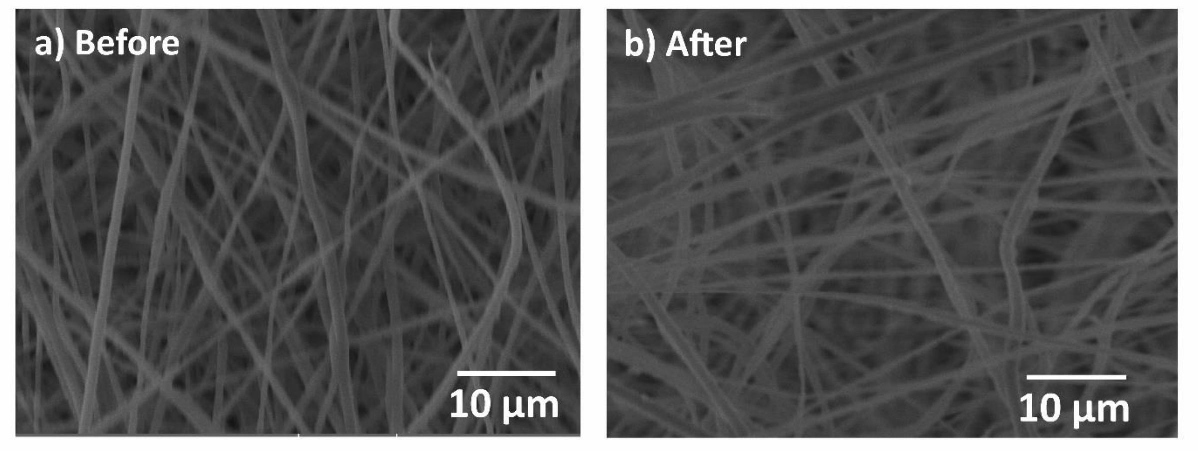

Figure S20. SEM image of 5 wt% ZIF-8-Cl/PVDF fiber (a) before and (b) after the durability test.

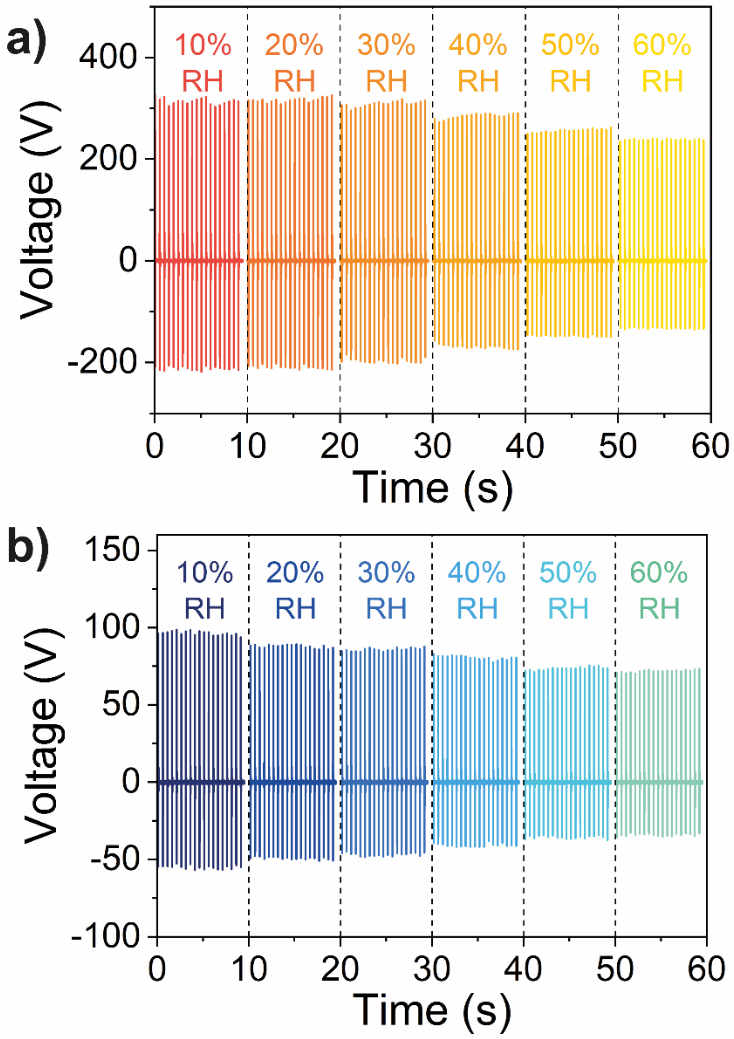

Figure S21. The triboelectric voltage output of (a) 5 wt% ZIF-8-Cl/PVDF fiber and (b) neat PVDF fiber under different relative humidity. Aluminum was used as the counter electrode.

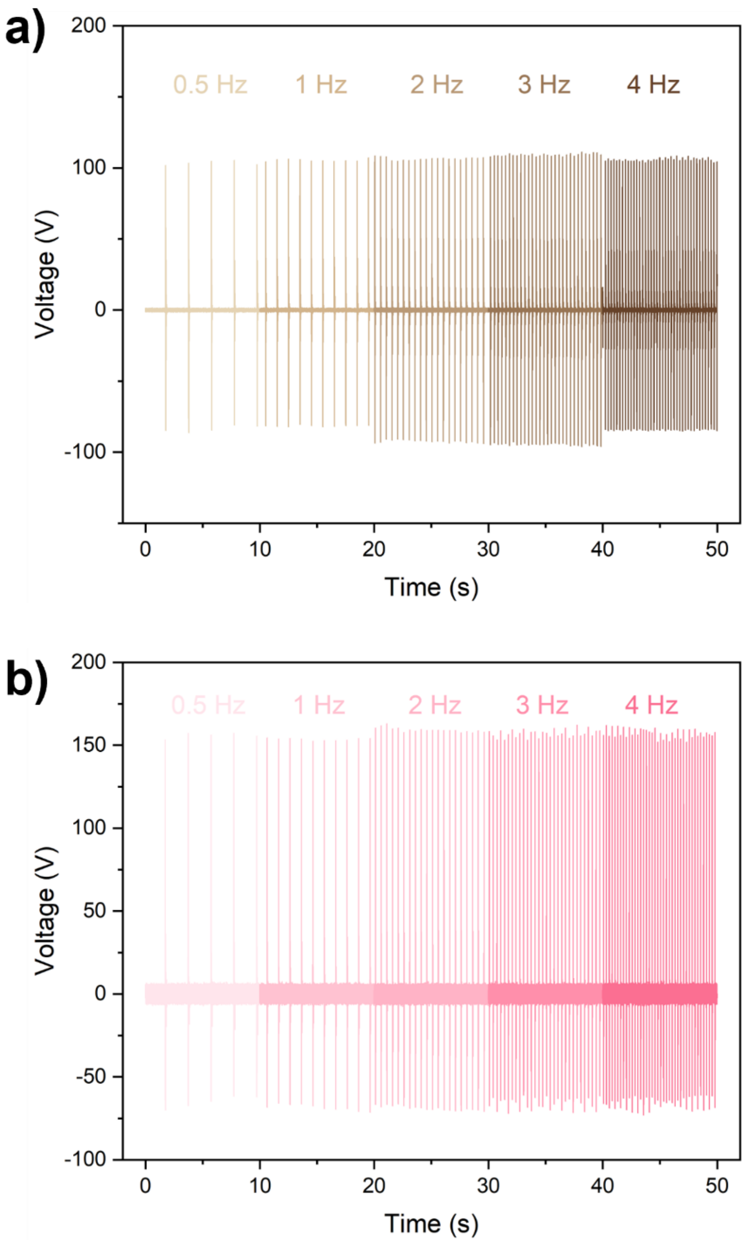

Figure S22. a) Open-circuit voltage at different operating frequencies for a) 5 wt% ZIF-8-CH_3/PVDF based TENG and b) 5 wt% ZIF-8-Br/PVDF based TENG.

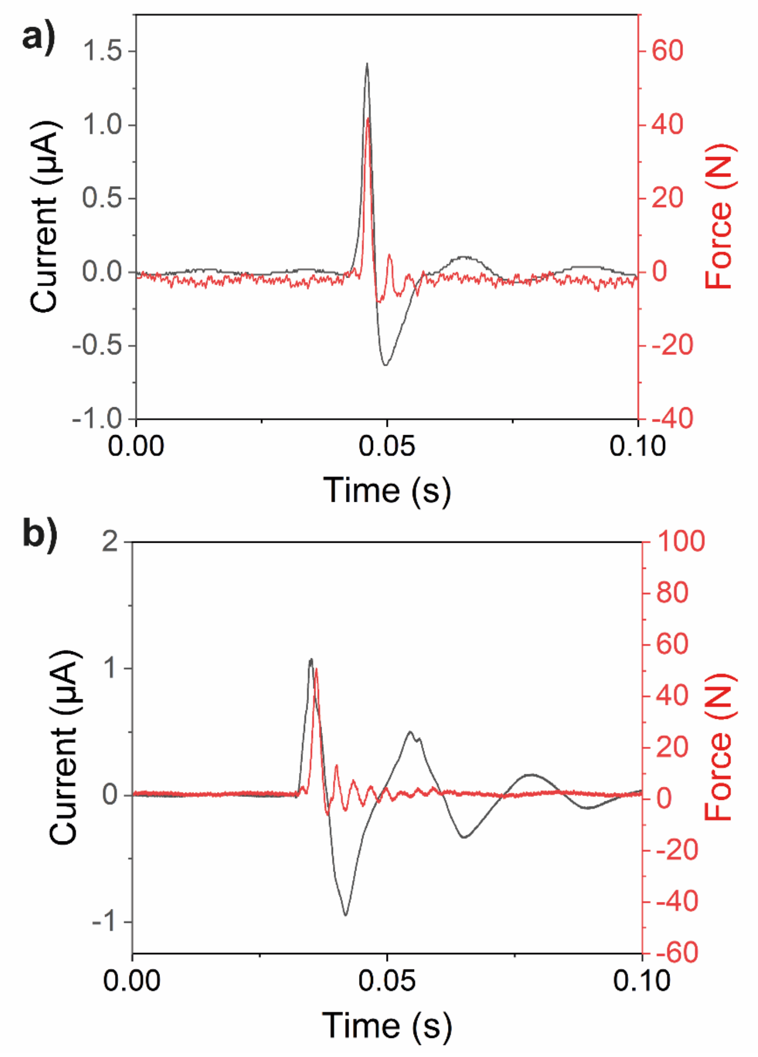

Figure S23. Relationship between the force applied on a) 5 wt% ZIF-8-Br/PVDF-based TENG and b) 5 wt% ZIF-8-CH_3/PVDF-based TENG and the output current tested on the same time scale.

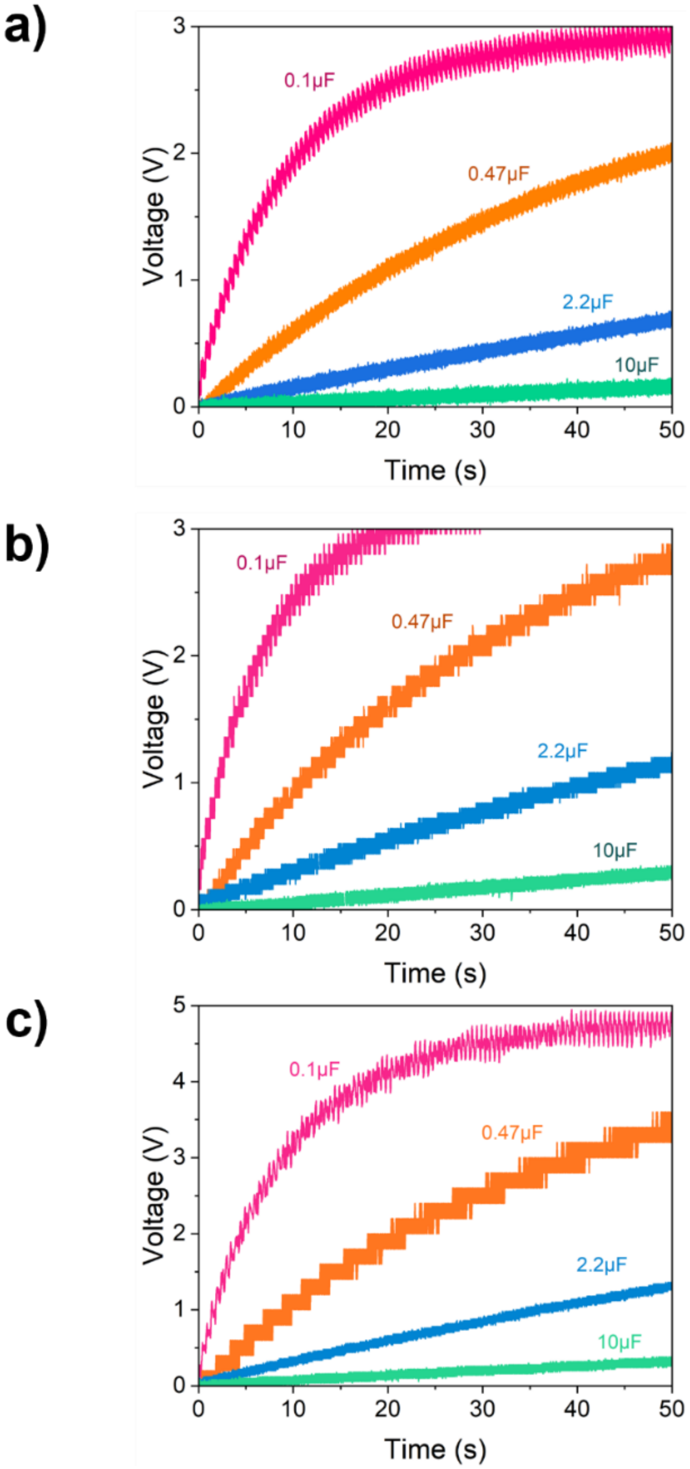

Figure S24. Charging of 0.1, 0.47, 2.2, and 10 μF capacitors by a) 5 wt% ZIF-8-CH_3/PVDF-based TENG, b) 5 wt% ZIF-8-Br/PVDF-based TENG, and c) 5 wt% ZIF-8/PVDF-based TENG operating at 2 Hz.

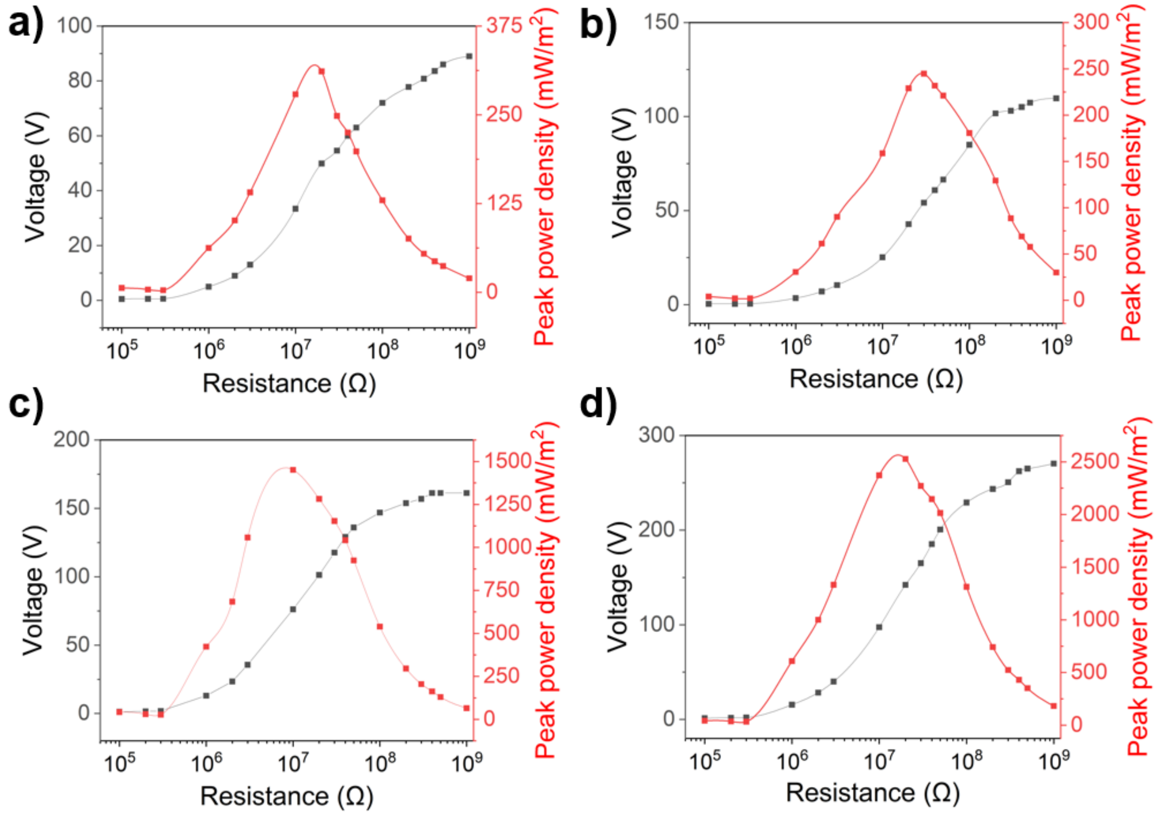

Figure S25. Peak voltage and power density as a function of load resistance for a) PVDF-based TENG, b) 5 wt% ZIF-8-CH_3/PVDF-based TENG, c) 5 wt% ZIF-8-Br/PVDF-based TENG, and d) 5 wt% ZIF-8-Cl/PVDF-based TENG.

Table S1. Comparison of triboelectric performance between PVDF-based TENG devices incorporating MOFs or other materials as fillers.

Tribo-positive	Tribo-negative	Area	Voltage	Current	Power density	Application	Ref.
Nylon-66	Ni-MOF/ PVDF	2.5 × 2.5 cm^2	45 V	0.77 μA	1.04 mW/m^2	self-powered pulse sensor	1
Cellulose aerogel/ Ni-HITP	PVDF	5.5 cm diameter circle	80 V	1.6 μA	11.5 mW/m^2	self-powered air filter	2
Al	MIL-101(Cr) /PVDF	2 × 2 cm^2	536 V	21.7 μA	8712 mW/m^2	smart home control	3
Al	MIL-53 (Ni)/ PVDF	2 × 3 cm^2	15 V	1.5 μA	3.1 mW/m^2	human motion detection	4
Al	$CsPbI_3$/ PVDF	3 × 2.5 cm^2	377 V	43 μA	4107 mW/m^2	Moisture Monitoring	5
AgNW	tourmaline/ PVDF	2 × 2 cm^2	267 V	0.7 μA	107 mW/m^2	Energy harvesting	6
Al	C_{60}/ PVDF	2 × 2 cm^2	~80 V	~8 μA	705 mW/m^2	Power electronic device	7
ITO	$CoFe_2O_4$/ PVDF	2 × 2 cm^2	17.2 V	2.27 μA	90.3 mW/m^2	Harvest droplet energy	8
Al	ZIF-8-Cl/ PVDF	2 × 2 cm^2	312 V	4.9 μA	2540 mW/m^2	Rotational energy harvesting	This work

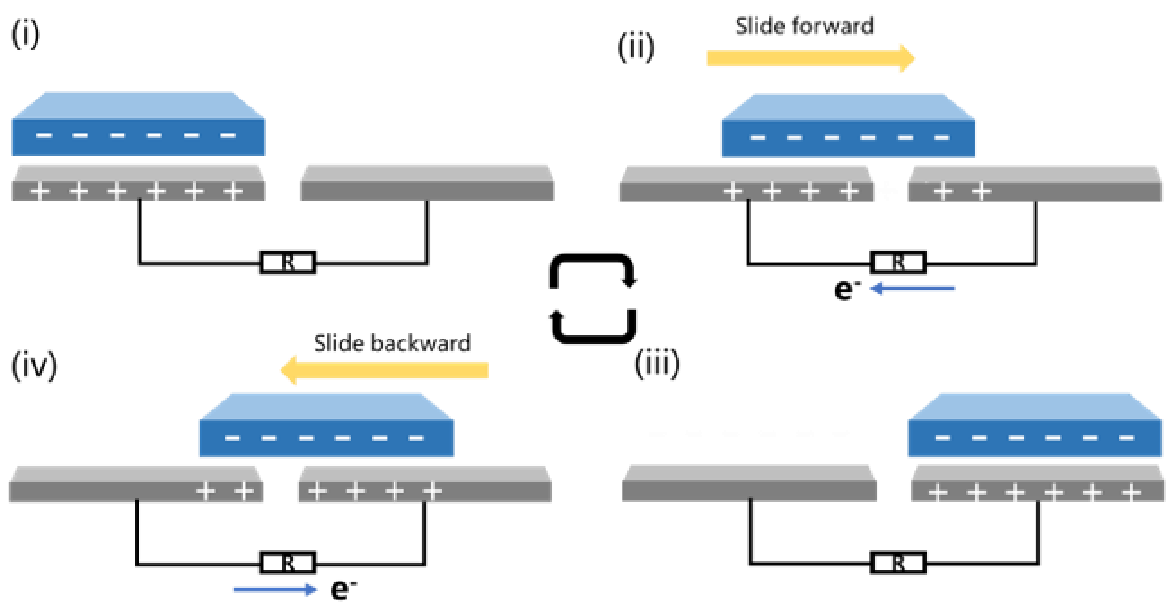

Figure S26. Schematic diagram of the proposed working mechanism (i-iv) of a free-standing mode TENG, based on ZIF-8-X/PVDF nanocomposite. The ZIF-8-X/PVDF material is initially rubbed with a copper sheet to accumulate saturated negative charges on the surface. When the negatively charged material is placed above the electrode, the potential difference induces a positive charge on the left electrode. As the nanocomposite moves to the right, the inductive potential difference shifts from the left to the right electrode, driving the electrons to flow through the external circuit. The reversed flow of electrons occurs when the material moves in opposite direction. Through periodic movement between the two electrodes, an alternating current is generated.

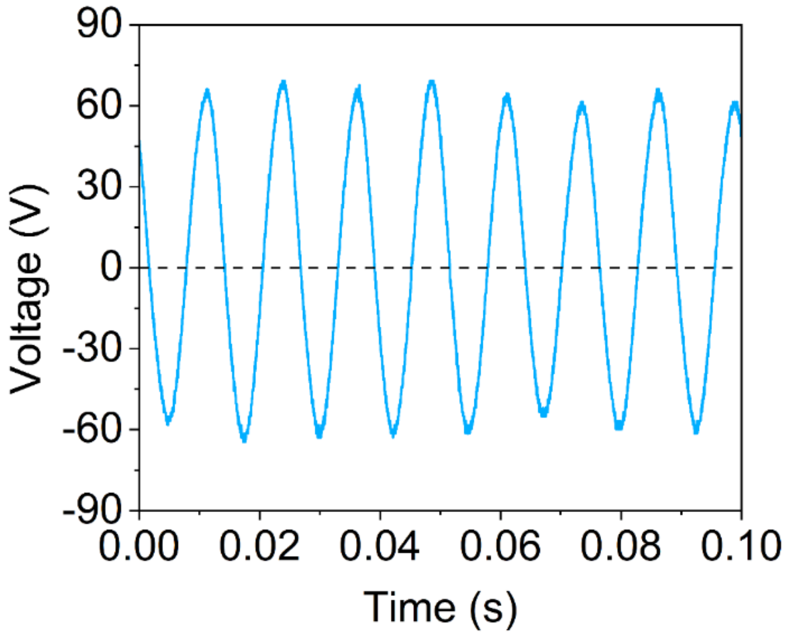

Figure S27. a) Open circuit voltage profile of the ZIF-8-Cl/PVDF-based non-contacting TENG, under a rotation speed of 600 rpm. Each rotation induces changes in potential by the 8 fan blades, therefore generating an AC output of 80 Hz.

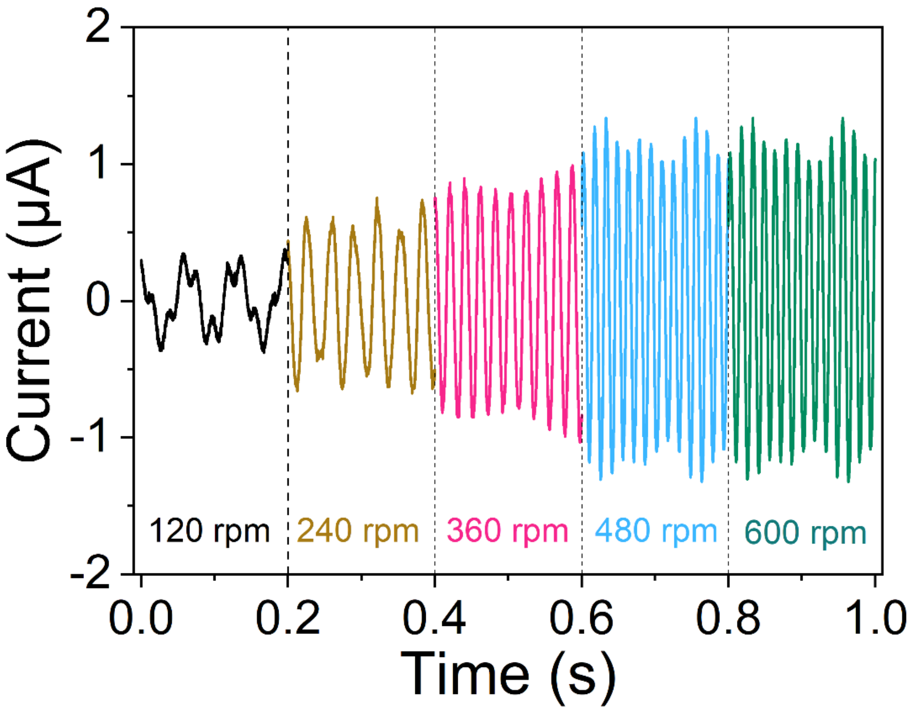

Figure S28. Current output of ZIF-8-Cl/PVDF-based non-contacting TENG at different rotational speeds from 120 rpm to 600 rpm.

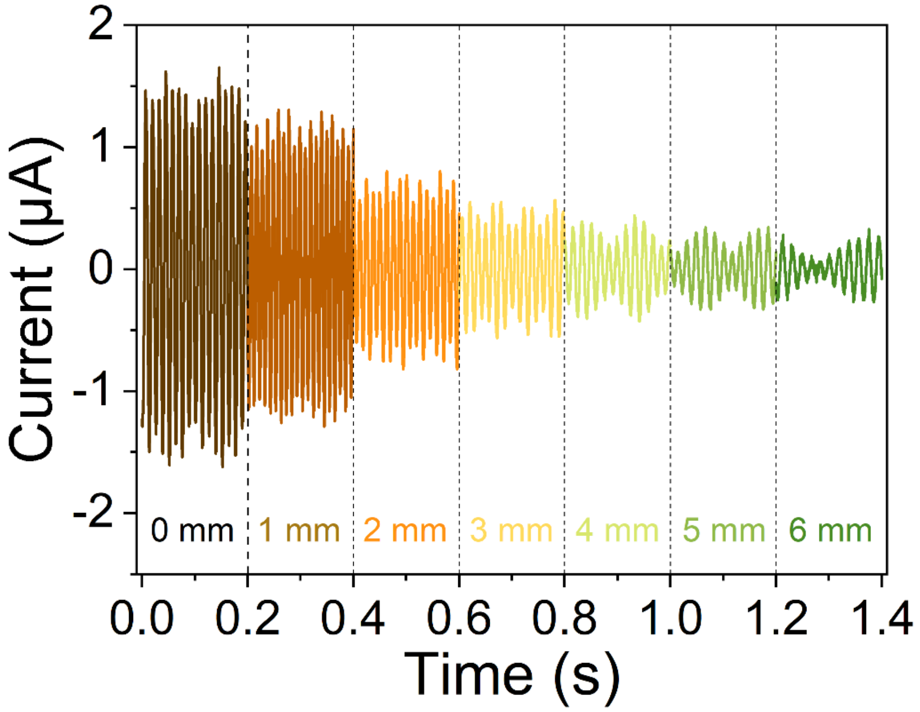

Figure S29. Current output of ZIF-8-Cl/PVDF-based non-contacting TENG with the same rotational speed but by varying gap distance between the rotor and stator from 0 and 6 mm.

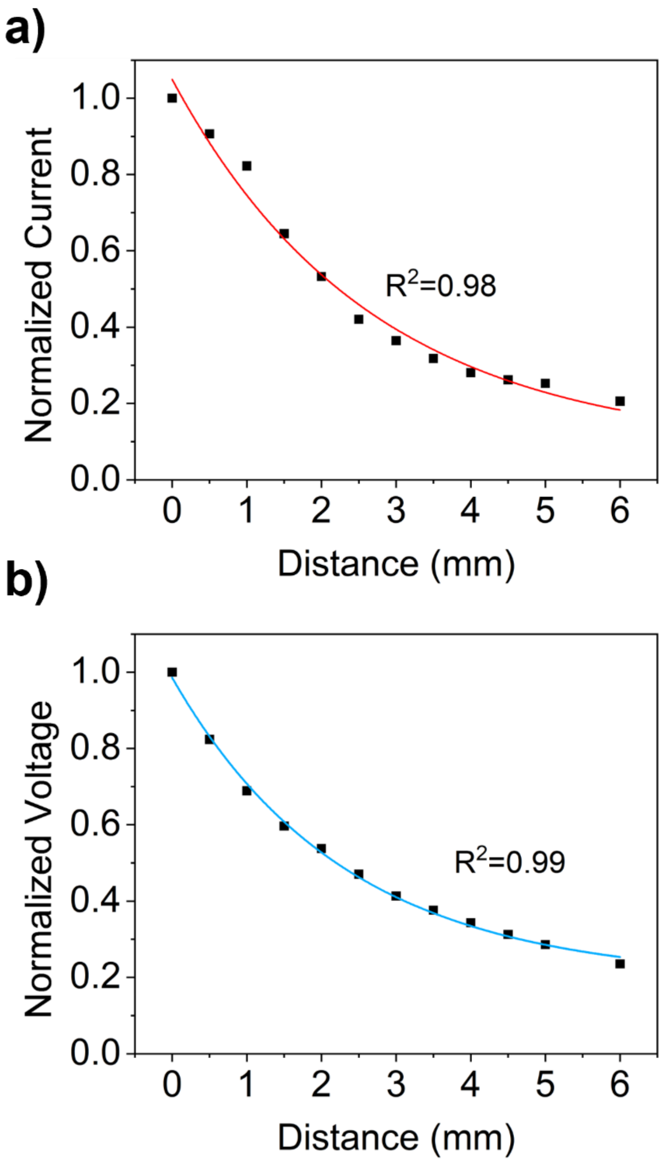

Figure S30. a) Inverse relationship between the voltage generated by ZIF-8-Cl/PVDF-based non-contacting TENG and the gap distance. b) Inverse relationship between the current generated by ZIF-8-Cl/PVDF-based non-contacting TENG and the gap distance.

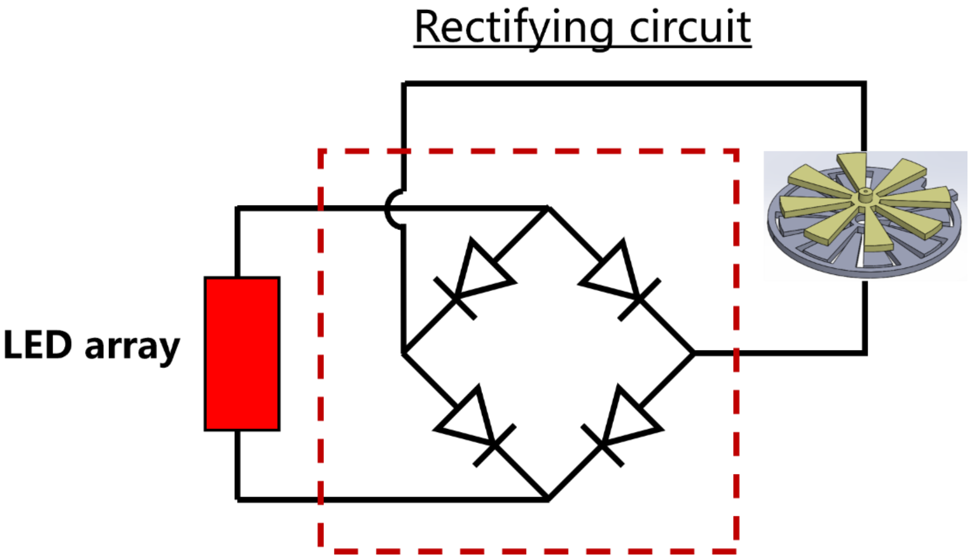

Figure S31. Schematic of an electrical circuit designed for the illumination of LEDs by ZIF-8-Cl/PVDF-based non-contacting TENG.

Video S1. Demonstration of a non-contacting free standing mode TENG based on ZIF-8-Cl/PVDF composite continuously powering a LED array.

References

(1) Das, N. K.; Ravipati, M.; Badhulika, S. Nickel Metal-Organic Framework/PVDF Composite Nanofibers based Self-Powered Wireless Sensor for Pulse Monitoring of Underwater Divers via Triboelectrically Generated Maxwell- Displacement Current. *Adv. Funct. Mater.* **2023**, *33* (37), 14.

(2) Fu, Q.; Liu, Y. H.; Liu, T.; Mo, J. L.; Zhang, W. L.; Zhang, S.; Luo, B.; Wang, J. L.; Qin, Y.; Wang, S. F.; Nie, S. X. Air-permeable cellulosic triboelectric materials for self-powered healthcare products. *Nano Energy* **2022**, *102*, 10.

(3) Sohn, S. H.; Choi, G. J.; Park, I. Metal-organic frameworks-induced Self-Poling effect of polyvinylidene fluoride nanofibers for performance enhancement of triboelectric nanogenerator. *Chem. Eng. J.* **2023**, *475*, 12.

(4) Sasmal, A.; Senthilnathan, J.; Arockiarajan, A.; Yoshimura, M. Two-Dimensional Metal-Organic Framework Incorporated Highly Polar PVDF for Dielectric Energy Storage and Mechanical Energy Harvesting. *Nanomater.* **2023**, *13* (6).

(5) Mondal, S.; Maiti, S.; Paul, T.; Poddar, S.; Das, B. K.; Chattopadhyay, K. K. CsPbI3–PVDF Composite-Based Multimode Hybrid Piezo-Triboelectric Nanogenerator: Self-Powered Moisture Monitoring System. *ACS Appl. Mater. Interfaces* **2024**, *16* (7), 9231-9246.

(6) Pan, J.; Jin, A. Improvement of Output Performance of the TENG Based on PVDF by Doping Tourmaline. *ACS Sustain. Chem. Eng.* **2024**, *12* (5), 2092-2099.

(7) Sim, D.-J.; Choi, G.-J.; Sohn, S.-H.; Park, I.-K. Electronegative polyvinylidene fluoride/C60 composite nanofibers for performance enhancement of triboelectric nanogenerators. *J. Alloy Compd.* **2022**, *898*, 162805.

(8) Vu, D. L.; Ahn, K. K. Triboelectric Enhancement of Polyvinylidene Fluoride Membrane Using Magnetic Nanoparticle for Water-Based Energy Harvesting. *Polymers* **2022**, *14* (8).
